# Crystal Structure of the Human Cytomegalovirus Glycoprotein B

**DOI:** 10.1371/journal.ppat.1005227

**Published:** 2015-10-20

**Authors:** Heidi G. Burke, Ekaterina E. Heldwein

**Affiliations:** Department of Molecular Biology and Microbiology and Graduate Program in Molecular Microbiology, Sackler School of Graduate Biomedical Sciences, Tufts University School of Medicine, Boston, Massachusetts, United States of America; Institut Pasteur, FRANCE

## Abstract

Human cytomegalovirus (HCMV), a dsDNA, enveloped virus, is a ubiquitous pathogen that establishes lifelong latent infections and caused disease in persons with compromised immune systems, e.g., organ transplant recipients or AIDS patients. HCMV is also a leading cause of congenital viral infections in newborns. Entry of HCMV into cells requires the conserved glycoprotein B (gB), thought to function as a fusogen and reported to bind signaling receptors. gB also elicits a strong immune response in humans and induces the production of neutralizing antibodies although most anti-gB Abs are non-neutralizing. Here, we report the crystal structure of the HCMV gB ectodomain determined to 3.6-Å resolution, which is the first atomic-level structure of any betaherpesvirus glycoprotein. The structure of HCMV gB resembles the postfusion structures of HSV-1 and EBV homologs, establishing it as a new member of the class III viral fusogens. Despite structural similarities, each gB has a unique domain arrangement, demonstrating structural plasticity of gB that may accommodate virus-specific functional requirements. The structure illustrates how extensive glycosylation of the gB ectodomain influences antibody recognition. Antigenic sites that elicit neutralizing antibodies are more heavily glycosylated than those that elicit non-neutralizing antibodies, which suggest that HCMV gB uses glycans to shield neutralizing epitopes while exposing non-neutralizing epitopes. This glycosylation pattern may have evolved to direct the immune response towards generation of non-neutralizing antibodies thus helping HCMV to avoid clearance. HCMV gB structure provides a starting point for elucidation of its antigenic and immunogenic properties and aid in the design of recombinant vaccines and monoclonal antibody therapies.

## Author Summary

Human cytomegalovirus (HCMV) establishes lifelong infection in a majority of the world’s population and causes disease in neonates and the immunocompromised patients such as organ transplant recipients or persons with AIDS. There is no vaccine against HCMV, and current HCMV antivirals are toxic and an increasing prevalence of resistance. Glycoprotein B (gB), displayed on the viral surface is a major viral immunogen and is necessary for viral penetration into cells. The crystal structure of gB reported here provides a detailed 3D map of gB. A thick glycan layer covers a large surface area, which may explain why anti-gB neutralizing antibodies are relatively rare. The structure is expected to aid in the development of a HCMV vaccine and monoclonal antibody therapies.

## Introduction

Herpesviruses are double-stranded DNA, enveloped viruses that cause lifelong latent infections. These viruses are divided into three subfamilies, alpha-, beta-, and gammaherpesviruses. Human cytomegalovirus (HCMV), a betaherpesvirus, is prevalent in the United States with 50–80% of adults being seropositive by the age of 40 [1]. While HCMV establishes lifelong latent infections, its reactivations are typically suppressed by competent immune systems. However, HCMV is capable of causing disease in the immunocompromised including such symptoms as gastrointestinal ulceration, hepatitis, pneumonitis or retinitis in solid organ transplant patients [2] and retinitis in patients with AIDS, which can lead to blindness [2]. HCMV is also a leading cause of congenital viral infections in newborns where it can cause permanent defects such as deafness, blindness, epilepsy, mental retardation and microcephaly [3]. The antiviral ganciclovir and immunoglobulin from seropositive individuals (CMVIG) have been used for treatment and prophylaxis [4], with ganciclovir being more effective and the standard of care. Unfortunately, ganciclovir has associated toxicity and cannot be administered to some patients such as pregnant women [5]. Additionally, rising resistance to ganciclovir is a major concern [6, 7]. A better understanding of the immune response elicited by gB is needed to generate improved neutralizing monoclonal antibody (mAb) therapeutics and recombinant protein vaccines.

HCMV genome encodes many glycoproteins. Seven of these: gB, gH, gL, gO, UL128, UL130 and UL131, are critical for cell entry (reviewed in [8]). gH/gL/gO (trimer) and gH/gL/UL128/UL130/UL131 (pentamer) determine cellular tropism and likely function as receptor-binding proteins [9–11]. gB is essential for entry into all cell types and is conserved among herpesviruses [8]. Binding of gB to cellular integrins or PDGFR has been proposed to initiate cellular signaling cascades necessary for viral internalization [12, 13]. By analogy with its homologs from other herpesviruses, such as Herpes Simplex virus and Epstein-Barr virus, gB is also thought to function as a fusogen [8].

Viral fusogens mediate the merger of the viral envelope and host membrane during entry and cell spread by undergoing a series of conformational changes from the prefusion to the postfusion form, mapped out for several viral fusogens (reviewed in [14]). The energy released during this refolding is thought to drive membrane fusion [14]. The conformational pathway has not yet been mapped for any of gB homologs and may have unique features due to the reliance on additional viral proteins for function [15]. The available crystal structures of gB ectodomains from HSV and EBV show their postfusion forms [16, 17], while their prefusion conformations have not yet been characterized. The postfusion structures of HSV and EBV gB share a structural similarity with vesicular stomatitis virus (VSV) glycoprotein G [18] and baculovirus gp64 [19]. Together, these four proteins form the recently discovered class III of viral fusogens [20].

**Fig 1:**
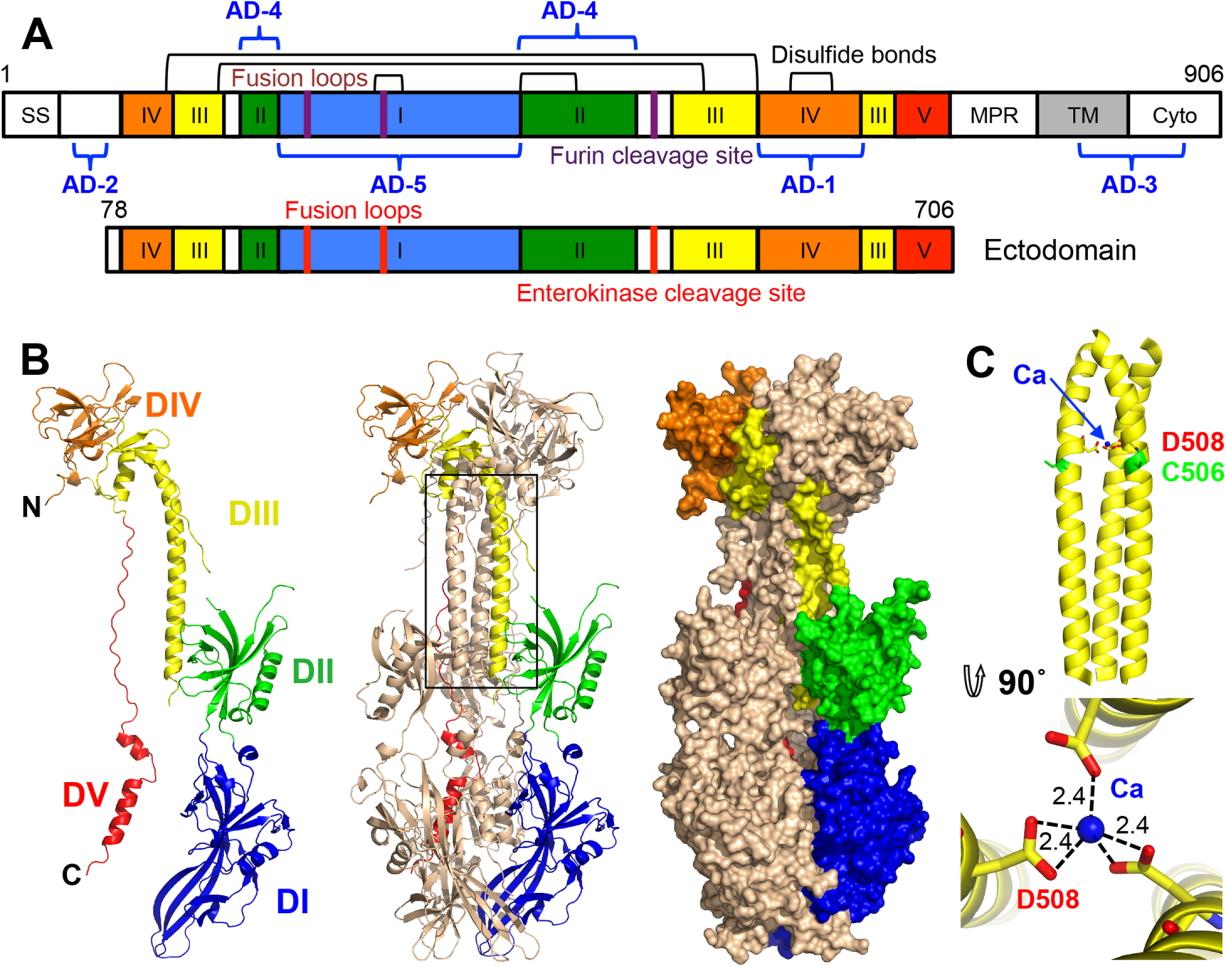
HCMV gB ectodomain structure. (A) Schematic representation of the full-length HCMV gB (top) and the crystallized construct, gB78-706-7M-E (bottom). Disulfide bonds are represented as black brackets, antigenic domains (AD-1-5) are indicated in blue brackets, and mutations are shown using red bars. Structural domains, are colored as follows: domain I = blue, II = green, III = yellow, IV = orange, V = red, as in [16, 17]. SS = signal sequence, MPR = membrane proximal region, TM = transmembrane domain, and Cyto = cytoplasmic domain. Numbers denote construct boundaries. (B) The crystal structure of the HCMV gB ectodomain is shown as a protomer and a trimer in cartoon representation as well as a trimer in surface representation. Chain B is colored by domain as in (A). (C) Side and top down view of the coiled coil in DIII with a coordinated calcium ion (CA) (blue sphere). Side chains of D508 (yellow) with carboxyl oxygens (red) and C506 (green) are also shown. Dashed lines indicate distances between the carboxyl oxygens in D508 and the calcium ion. All structure figures were made in Pymol (http://www.pymol.org).

gB is about 900 amino-acid long (HCMV: 906 aa; HSV-1: 904 aa; EBV: 857 aa) and contains a large ectodomain, a hydrophobic membrane-proximal region (MPR), a transmembrane domain (TM), and the intraviral (or cytoplasmic) domain (cytodomain) (Fig 1A). HCMV gB shares 24.2% and 30.2% identity with its HSV-1 and EBV homologs, respectively, within its ectodomain. Despite the relatively low sequence identity, the crystal structures of HSV-1 and EBV gB ectodomains are very similar. Both are spike-like trimers in which each protomer consists of 5 domains (DI-V) [16, 17]. DI, or fusion domain (FD), is composed of a pleckstrin homology domain (PHD) module and finger-like beta-sheet protrusions containing fusion loops at their tips. DII consists of another PHD. DIII, or core domain (CD), harbors the long helix that forms a central triple coiled coil within gB trimer. DIV, or crown domain (CRD) forms ear-like protrusions at the end of the spike distal from the fusion loops. Finally, DV is an extended polypeptide that spans nearly the entire length of the gB spike and fits into a long groove formed by DIII and DI of the two neighboring protomers.

gB (along with gH/gL and the pentamer, gH/gL/UL128/UL130/UL131) elicits a strong immune response in humans and induces the production of neutralizing antibodies, though most anti-gB Abs are non-neutralizing [21, 22]. gB is the most highly conserved among the HCMV entry glycoproteins and is required for entry into all cell types whereas the pentamer is necessary for entry into epithelial and endothelial cells but not fibroblasts. Therefore, gB may represent a better antigenic target for monoclonal immunoglobulin therapy. In particular, anti-gB IgG could protect human trophoblast progenitor cells (TBPCs), the precursors to placental cells, while anti-pentamer IgG could not [23].

Five antigenic sites, AD-1-5, where AD stands for antigenic domain, have been identified in HCMV gB (Fig 1A). AD-1 produces the strongest immune response yet largely generates non-neutralizing antibodies [21, 24] AD-2, located within the unresolved N terminus, is mildly immunogenic and has two sub-antigenic sites. Site I elicits only neutralizing antibodies while site II elicits only non-neutralizing antibodies [25], both are only mildly immunogenic with about 50% of HCMV-positive individuals producing anti-AD-2 antibodies [21, 25]. AD-3 is a linear epitope located within the cytodomain and does not generate neutralizing antibodies [26]. Recently identified AD-4 and AD-5 are highly immunogenic, produce only neutralizing antibodies, and are conserved [21]. Thus, AD-4, AD-5, and possibly AD-2 (site I) are the most promising candidates for production of therapeutic immunoglobulins.

Here, we determined the crystal structure of the gB ectodomain to 3.6-Å resolution. The structure of HCMV gB resembles the postfusion structures of HSV-1 and EBV homologs, making it a new class III viral fusogen. Despite structural similarities, each gB has a unique domain arrangement, demonstrating structural plasticity of gB that may accommodate virus-specific functional requirements. Large areas of the gB surface are shielded by glycans, which likely aid HCMV in evading the humoral immune response, helping explain the limited neutralization response against gB during natural HCMV infection [21]. The structure provides an important framework for elucidating the immunogenic determinants.

## Results

### Construct design, crystallization and structure determination

Previously, we showed that the purified ectodomain of HCMV gB formed rosette-like aggregates, in which gB molecules associated through their exposed fusion loops [27]. To eliminate aggregation, we had replaced four exposed hydrophobic residues within the fusion loops, Y155, I156, Y157, and W240, with their more hydrophilic HSV-1 counterparts. Although these four mutations reduced aggregation [27], they did not abolish it. Analysis of an HSV-1 gB-based HCMV gB homology model, described previously [27], suggested that the remaining hydrophobic residues L241 and Y242 within the second fusion loop and the nearby residue Y206 could be responsible for the residual aggregation (S1A Fig). To obviate aggregation, residues Y206 and Y242 were replaced with their HSV-1 counterparts while residue L241 was replaced with its EBV counterpart because HSV-1 has a hydrophobic residue, phenylalanine, at this position (S1 Fig). The resulting septuple mutant Y155G/I156H/Y157R/Y206H/W240A/L241T/Y242H (gB706-7M) was monodisperse (S2 Fig) and used in all subsequent work.

HCMV gB contains a furin cleavage site (residues 456–459) within the unstructured loop in DII that is partially cleaved during expression in mammalian cells [28, 29] (Fig 1A). Insect cells express furin-like proteases [30], and the gB706-7M mutant was partially cleaved during expression in Sf9 cells (S3 Fig). gB706-7M did not crystallize, and to obtain crystals, it was treated with low amounts of trypsin, a strategy that was successful in obtaining diffraction-quality crystals of HSV-1 gB ectodomain [16]. Although trypsin-cleaved gB706-7M crystallized, crystals clustered and diffracted poorly. N-terminal sequencing revealed that trypsin treatment, in addition to cleaving the unstructured loop in DII, resulted in cleavage that generated heterogeneous N termini starting at residues R51, R67, or Y78. To eliminate heterogeneity within the N terminus, we generated a construct that started at residue Y78 (gB78-706-7M). This construct crystallized with or without trypsin treatment. Although trypsin treatment improved the diffraction, residual trypsin (present despite its removal post cleavage) resulted in further cleavage during sample storage and crystallization (S3 Fig), which caused crystal degradation. To obviate complications due to non-specific trypsin cleavage, recombinant furin was used instead, but proved inefficient at cleaving gB (S3 Fig). Finally, the furin cleavage site was replaced with an enterokinase site (gB78-706-7M-E), which prevented cleavage during expression. Uncleaved gB78-706-7M-E (Fig 1A) was stable during storage (S3 Fig) and yielded crystals with the strongest diffraction, which suggested that both the removal of the N terminus of gB and the elimination of heterogeneous cleavage in the unstructured DII loop was beneficial.

A 3.6-Å resolution data set collected on a crystal of uncleaved gB78-706-7M-E was used to determine the structure of HCMV gB by molecular replacement. Both HSV-1 gB and EBV gB were tested as search models, but only EBV gB yielded a clear solution. There is one gB ectodomain trimer in the asymmetric unit. The use of 3-fold averaging, *de novo* model tracing, and extensive manual rebuilding using density-modified maps ensured that the resulting structure was minimally biased by the search model.

### Structure of the postfusion conformation of HCMV gB ectodomain

**Fig 2:**
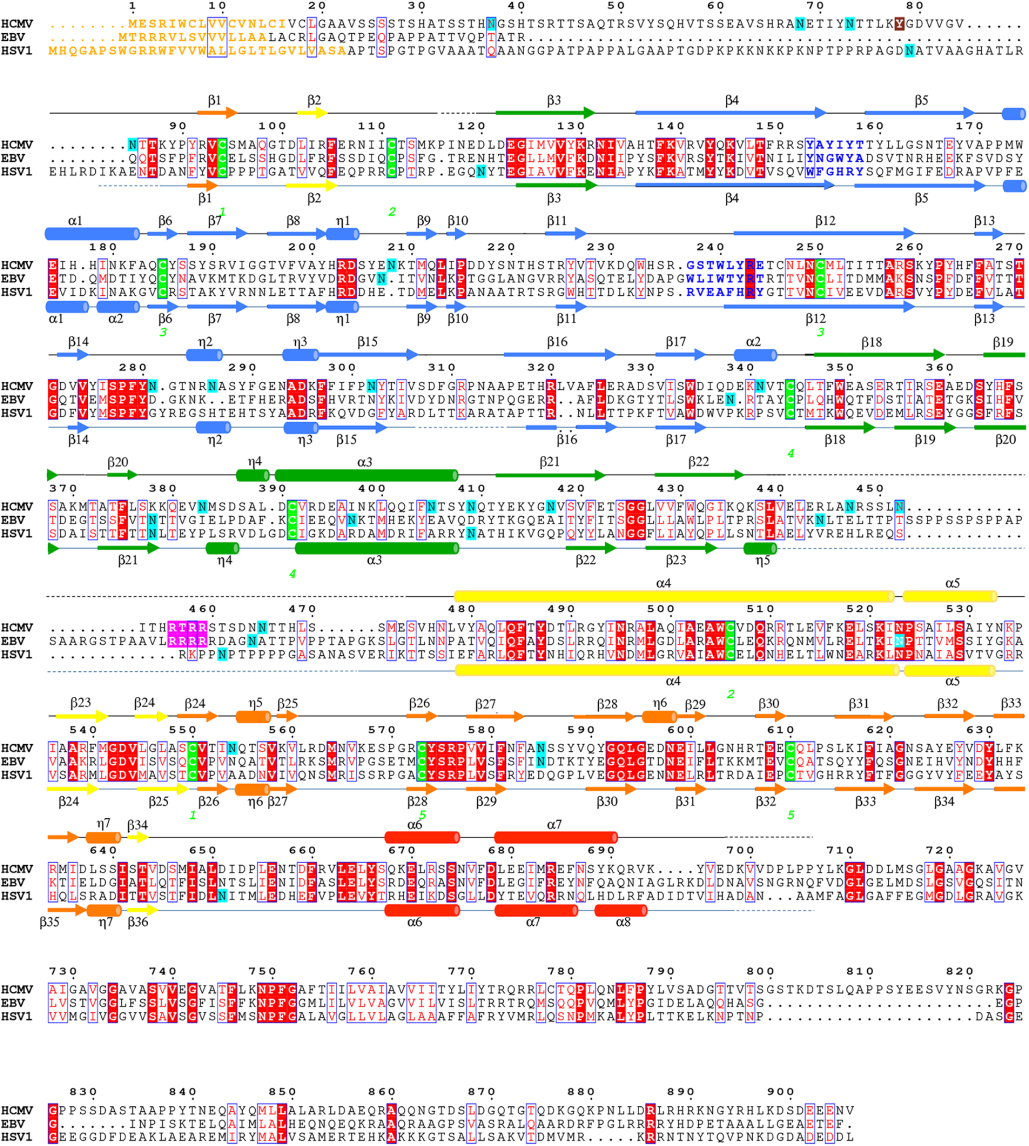
Multiple sequence alignment of gB homologs. Protein sequence alignment of HCMV (strain AD169), EBV (strain B958), and HSV-1 (strain KOS) (UniProtKB accession numbers P06473, Q777B0, P06437, respectively) was generated and analyzed using ClustalW2 [32] and ESPript 3.x [33]. The secondary structure of HCMV is displayed above the alignment and the secondary structure of HSV-1 (2GUM) [16] is displayed below the alignment. Secondary structure elements are colored by domain, as in Fig 1. Unresolved residues are denoted with dashed lines, signal sequences as orange letters and residues in fusion loops as blue letters. Cysteines participating in disulfide bonds are highlighted in green, furin cleavage sites, in pink, glycosylation sites, in cyan. First residue in the crystallized HCMV gB construct is highlighted in brown. Identical residues are shown as white text on red background, similar residues are shown as red text.

HCMV gB is an elongated trimer resembling a spike; each protomer consists of 5 domains (Figs 1B and 2) assigned based on the structure of the HSV-1 gB homolog [16]. Chain A contains residues 87–696 (unresolved 78–86, 117–120, 409–410, 435–475), chain B contains residues 86–697 (unresolved 78–85, 116–121, 439–474), and chain C contains residues 83–695 (unresolved 78–82, 117–118, 441–475). HCMV gB ectodomain contains eleven cysteines, ten of which are conserved. In the structure, these conserved cysteines form 5 disulfides leaving the non-conserved cysteine C246 unpaired. Previously, C246 was proposed to form a disulfide with a neighboring conserved cysteine C250 [31]. In the structure, C250 forms a disulfide bond with cysteine C185. Both C246 and C250 are located in strand β12 within the elongated fusion subdomain of DI and are unlikely to move into proximity.

A strong density found within the core of the central coiled coil, at the three-fold symmetry axis, was modeled as a Ca ion, which is coordinated by the side chains of D508 from the three gB protomers (Fig 1C). Residue D508 is located C-terminally to C506, which forms a disulfide with C111 from an extended region in the N terminus. This portion of the coiled coil is not expected to be unfolded in the prefusion form [34], by analogy with VSV G [35], which means that the Ca ion is unlikely to be involved in the conformational transition from the prefusion to the postfusion form. The Ca ion could potentially contribute to the stability of the coiled coil. Interestingly, the coordinating residue D508 is conserved among all CMV strains shown in Fig 2.

**Fig 3:**
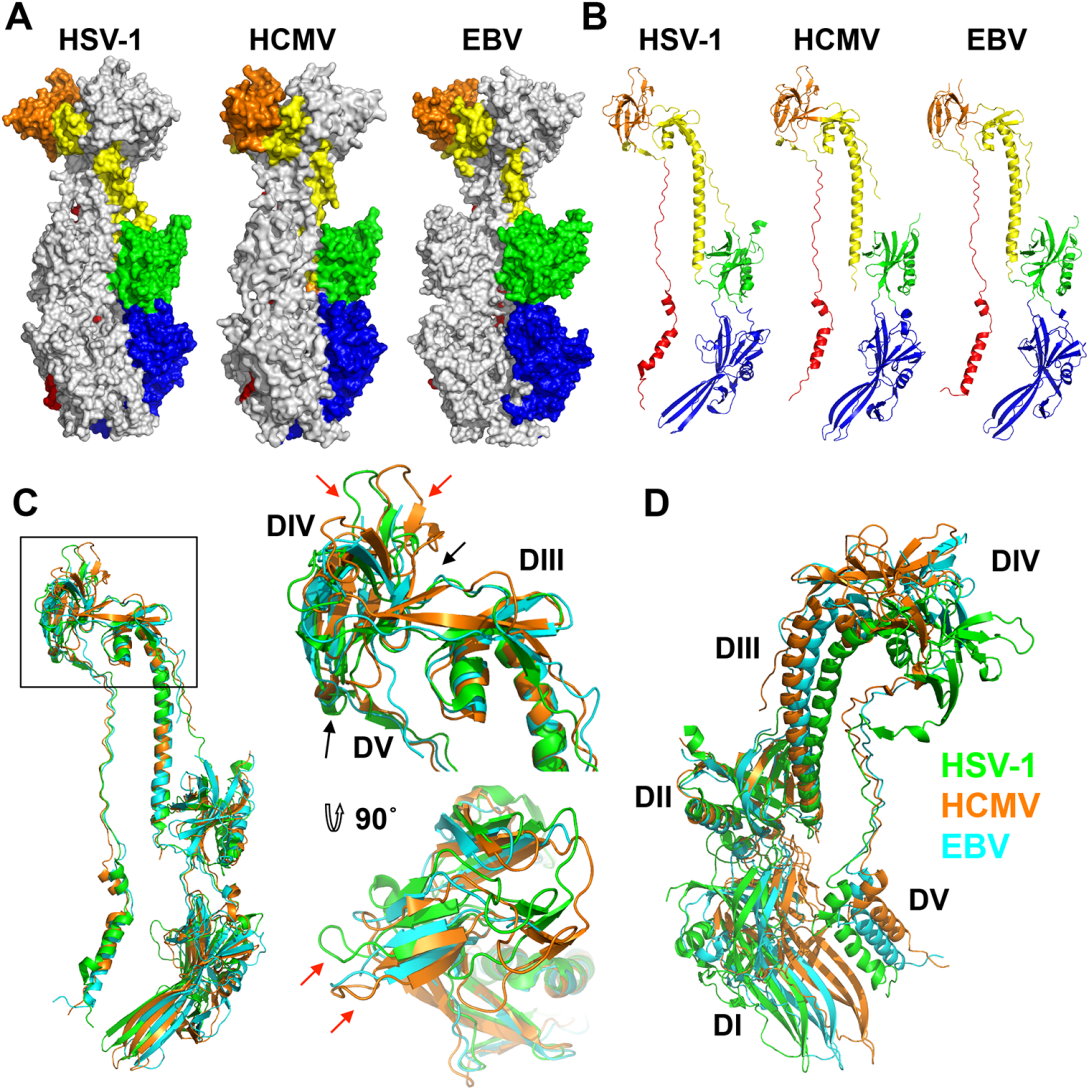
Structures of HCMV, HSV-1, and EBV gB ectodomains. The structures of HSV-1 (2GUM), HCMV (5CXF), and EBV (3FVC) gB ectodomains are aligned on the central trimeric helices and are displayed side-by-side as (A) trimers in surface and (B) protomers in cartoon representations. The domains I-V of each homologue are colored as in [16, 17]. (C) Single protomers (chain B in HSV-1 and HCMV) are aligned on the core helix α4. DIV and DIII are enlarged and shown from the side and top down. Red arrows indicate differences in the domain placement of the homologues, while black arrows indicate the hinge between domains. HSV-1 gB is colored in green, HCMV gB is colored in orange, and EBV gB is colored in cyan. (D) Single protomers (chain B in HSV-1 and HCMV) are aligned on DII, demonstrating the species-specific twist of each, using the same color scheme as in C.

The regions missing from the structure are likely conformationally flexible. The N terminus of each chain is unresolved, similarly to HSV-1 and EBV gB structures (Fig 3). Residues 441–473 make up the disordered loop in domain II that contains the furin cleavage site in the WT gB and the enterokinase cleavage site in the crystallized construct. Although this loop was left uncleaved in the crystallized construct, it remained flexible and unresolved in the structure.

HCMV is beginning to be appreciated as quasi species due to a high level of variability among strains [36, 37]. We aligned gB sequences from 60 clinical and laboratory isolates (S4 Fig) and mapped conservation patterns onto the structure (S5 Fig). Conservation scores of the analyzed strains ranged from 88.16 to 99.89%. The regions with the greatest diversity lie within the unstructured N terminus and the disordered DII loop, which are truncated or unresolved in the postfusion structure, respectively. Although the exact sequence of the furin cleavage site varies (RTRR, RTKR, or RAKR), and R468 was reported to be under positive selection [37], the furin cleavage site itself is completely conserved across all compared sequences. Although proteolytic processing of gB is dispensable for viral growth in culture [38], furin site conservation implies that it is beneficial to the virus for an as yet unknown reason. The least conserved region within the resolved structure is located within the plekstrin homology sub-domain of DI. The variable residues create a patch on the right face of this subdomain (S5 Fig). The next most variable region is nestled in the crown of DIV (S5 Fig).

### HCMV gB shares similar fold with HSV-1 and EBV homologs but has a distinct domain arrangement

**Fig 4:**
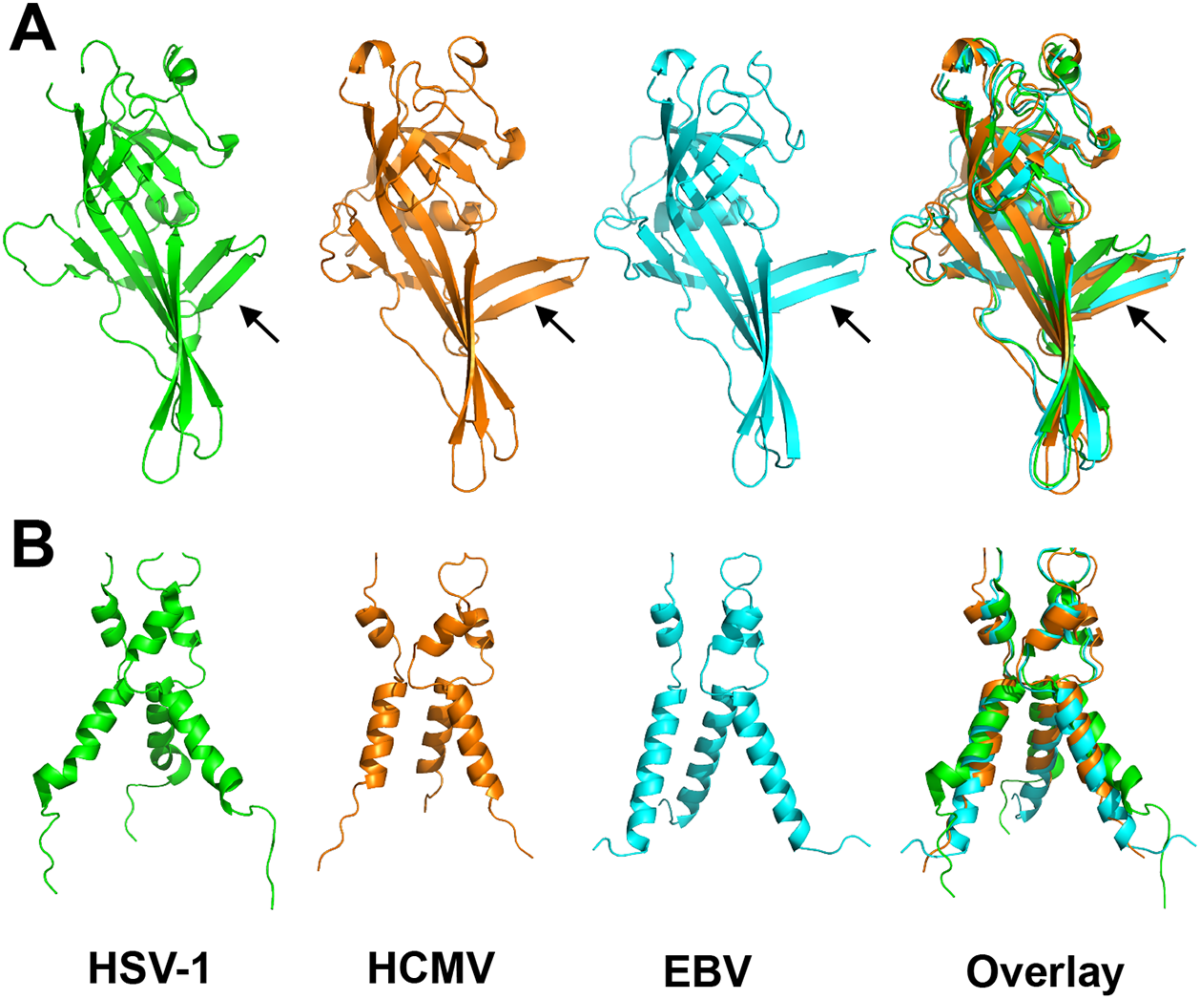
Alignment of gB homologs. (A) Individually aligned domains I of HSV-1 (2GUM) [16] in green, HCMV (5CXF) in orange, and EBV gB (3FVC) [17] in cyan are shown side by side and as an overlay. Residues used in alignments and RMSDs are listed in S1 Table. Orientation of the β hairpin β7-β8 (HCMV gB residues 188–201) is marked with an arrow. (B) Aligned trimers of domain V of each homolog are shown side by side and as an overlay. Colors of (B) are the same as in (A).

As anticipated from the 25–30% of sequence identity, the overall structure of the HCMV gB resembles the postfusion structures of HSV-1 and EBV gB ectodomains (Fig 3A and 3B). This similarity confirms that the HCMV gB structure represents its postfusion conformation and places HCMV gB among class III fusogens. Each of the 5 domains can be superimposed onto its counterpart (Fig 4, S6 Fig and S1 Table). Among the 5 domains, DII and DIII are the most similar (S6 Fig). DIV are also similar even though a number of loops in DIV of EBV gB are unresolved precluding detailed comparisons (S6 Fig). The fold of DI is also conserved with the exception of the β hairpin β7-β8 (residues 188–201 in HCMV gB), which adopts similar orientations in HCMV and EBV gB that differ from its orientation in HSV-1 (Fig 4A). Within the DV, helices α6 and α7 of HCMV gB DV differ from their counterparts in length and orientation (Fig 4B). Thus, in terms of the fold, domains that are closest to the (fused) membrane display the highest structural divergence.

Despite these local differences, the overall domain folds are very similar. By contrast, relative domain orientations are very different between HCMV, HSV-1, and EBV gB structures (Fig 3). The most obvious difference is within the hinge between DIII and DIV and the linker between DIV and DV, which positions DIV noticeably differently from that in HSV-1 and EBV relative to the gB “core”, DIII/DV (Fig 3C). Additional differences are found within the hinges linking DII and DIII, as well as DI and DII (Fig 3D). As the result, each gB protomer appears “twisted” in a unique way, which is apparent when the three structures are superposed on the conserved DII (Fig 3C and 3D). This architectural variation suggests structural plasticity that could have evolved to fine-tune gB to carry out virus-specific functions while preserving its conserved fusogenic role.

### Locations of motifs of potential functional importance

Several motifs of potential functional importance have been identified in HCMV gB. A peptide corresponding to residues 678–694 inhibited cell entry of several HCMV strains [39]. The structure helps explain this inhibitory activity because residues 678–694 form helix α7 in the extended DV, which forms the outer layer of the trimeric postfusion hairpin (Fig 1B). Analogous peptides derived from the outer layer of the postfusion hairpin in other viral fusogens block fusion by binding to the extended intermediate and preventing its refolding into a trimeric hairpin ((reviewed in [40, 41]). The HCMV peptide 678–694 may similarly inhibit fusion by blocking the formation of the postfusion form.

HCMV cannot enter cells deficient in β1 integrin [12], which suggests that HCMV binding to β1 integrins is necessary for entry into fibroblasts. It has been proposed that β1 integrin binding is mediated by a disintegrin-like motif within HCMV gB [12]. A peptide derived from this motif inhibited HCMV entry into fibroblasts [12] while a larger gB fragment immunoprecipitated β1 integrin [42]. A disintegrin-like motif (RX_5-7_DLXXF/L) was first identified in several ADAM (a disintegrin and metalloproteinase) family members [43] and is distinct from the classic integrin-binding RGD/KGD motif. In the HCMV gB structure, the proposed disintegrin-like motif does not form a domain but maps instead to the extended polypeptide that belongs to DIII and DIV. Residues identified as important for β1 integrin binding in ADAMs, except for D101, are buried (S7 Fig). Although we cannot exclude the possibility that these residues are exposed in the prefusion structure, how the proposed disintegrin-like motif in HCMV gB participates in binding to β1 integrins is currently unclear. Unfortunately, what the disintegrin-like motif in ADAMs look like is unknown because the only available crystal structure of a disintegrin domain is that of ADAM10, which lacks this motif [43].

### HCMV gB is extensively glycosylated

Unlike HSV-1 and EBV, HCMV gB ectodomain is extensively glycosylated, with eighteen predicted N-linked glycosylation sites in strain AD169 [27]. Within gB78-706, fifteen asparagines are predicted to be glycosylated. Of these, four are located within the unresolved regions of the polypeptide (N85, N447, N542, and N465) but all of the remaining eleven sites contain glycans in the structure (even though at some sites, the glycans are not ordered in all three polypeptides). Most glycans, with one exception, are unresolved beyond the first two NAG moieties due to their flexibility. Only glycan at residue N208 is well ordered with clear density for the mannose moieties beyond the branching point ((Man4-GlcNAc2-N-Asn)) (Fig 4). This glycan packs against the protein, with the second NAG stacking against the side chain of W174, which explains why it is well ordered in the crystal. Sequence alignment of HCMV gB sequences from 60 strains revealed that seventeen out of eighteen N-linked glycosylation sites are completely conserved (S4 Fig). The glycosylation site at N37 in AD169 is not conserved, while strains Towne, Merlin, and related clinical isolates contain a potential glycosylation site at N456, which is absent from AD169 strain.

**Fig 5:**
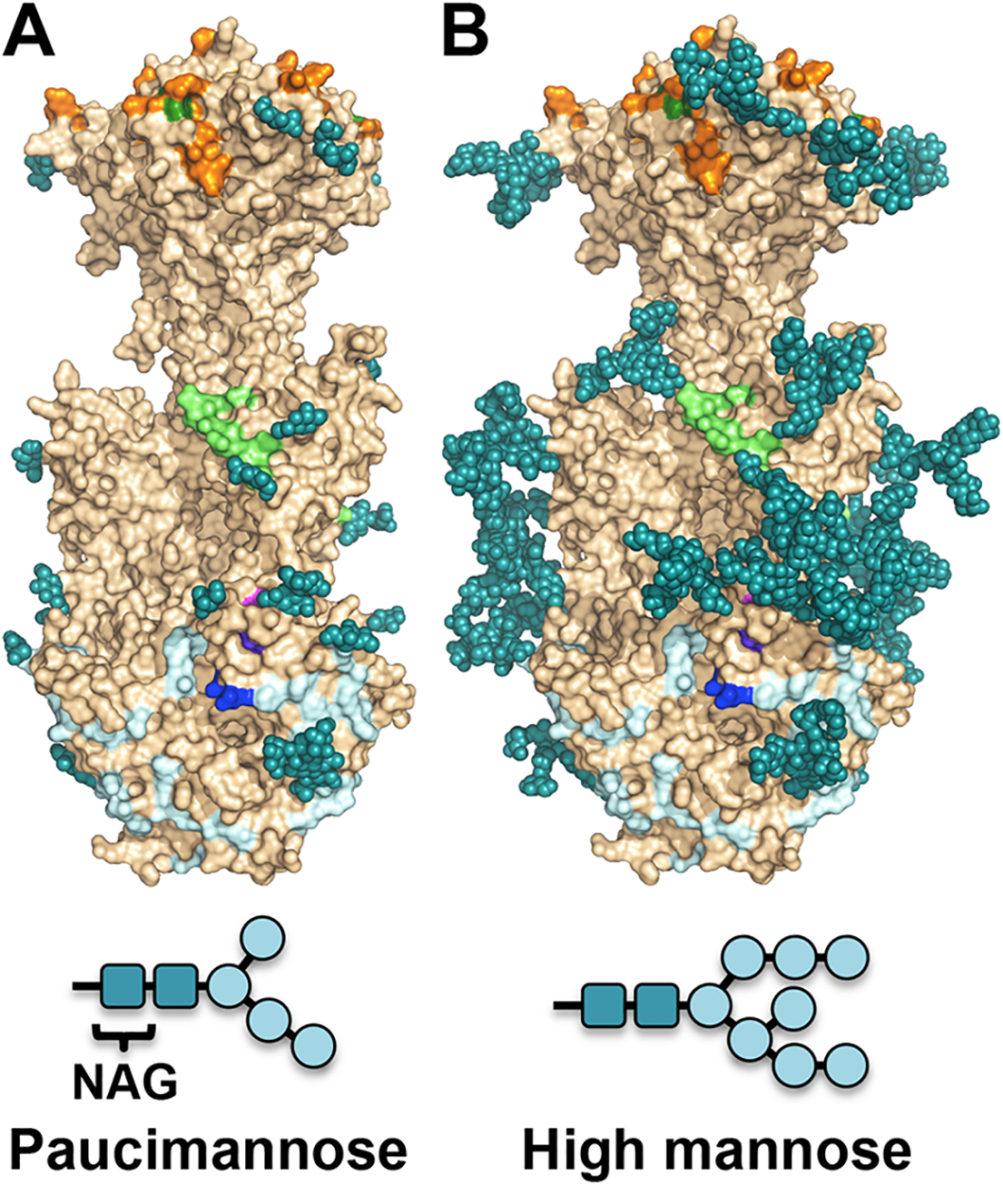
Observed glycosylation and fully-glycosylated model of HCMV gB. (A) HCMV gB is shown in surface representation in wheat. Glycans observed in the structure are shown in space-filled representation in teal. Residues shown to be important for antibody binding are colored as in Fig 6. (B) To obtain a glycosylation model representative of mammalian glycosylation, high-mannose type glycans (Man8-GlcNAc2-N-Asn) were modeled onto the structure of gB. Paucimannose and high-mannose are shown schematically.

The major processed N-glycan produced by insect cells is a highly trimmed paucimannose (Man3-GlcNAc2-N-Asn) (reviewed in [44]). To show what gB may look like on the viral surface, we modeled high-mannose type glycans to generate a fully-glycosylated model of gB (Fig 5). The model shows that much of the gB surface is shielded by a thick glycan layer. Similar glycan shields have been observed in HIV Env [45], Ebola gp [46], and EBV gp350 [47]. DII is the most heavily glycosylated, with four glycosylation sites in the structure, N383, N405, N409, and N417, plus three predicted glycosylation sites within its unresolved loop, N447, N452, and N465. Four out of five glycosylation sites in DI, N281, N286, N302, and N341, are within its upper pleckstrin homology subdomain. Only one glycosylation site, N208, is within the fusion subdomain of DI, and as described above, this glycan packs against the protein surface and points away from the fusion loops, which is consistent with the need for the fusion loops to be exposed for insertion into the membrane. Within DIV, there are two glycosylation sites, N555 and N585, which are located next to each other at the apexes of the trimer “crown” leaving the top of the crown exposed. The core of the gB trimer, consisting of DIII and DV, is not glycosylated.

### Neutralizing antibodies and epitopes

**Fig 6:**
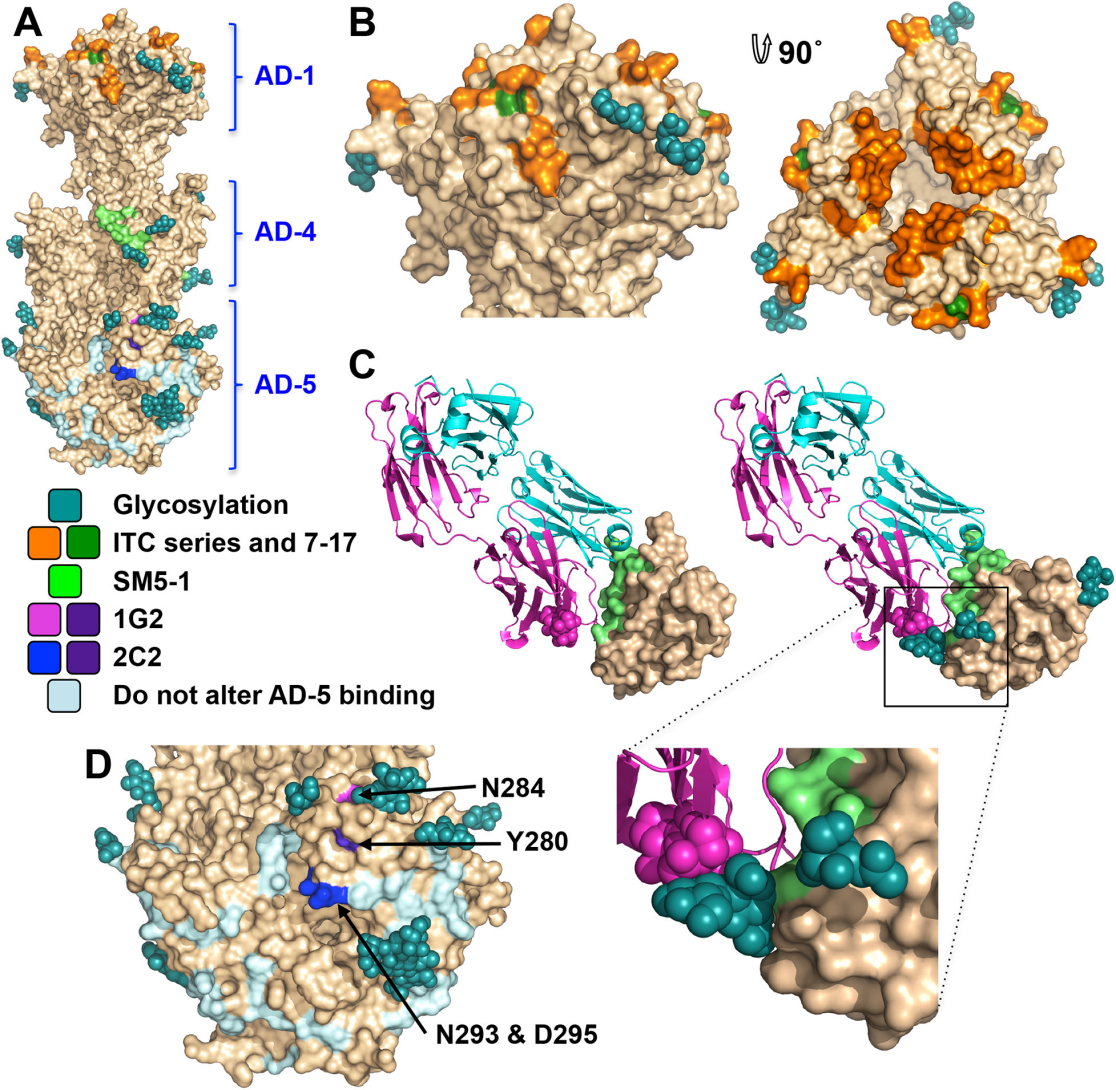
Neutralizing antibody epitopes. (A) The overall view of the epitopes on the surface of gB. (B) Side and top view of domain IV (AD-1). Residues shown to decrease binding of anti-AD-1 Abs are shown in orange. Those specific to neutralizing AD-1 Abs (G595 and F632) are colored forest green. (C) Domain II (AD-4) in complex with a human neutralizing Ab, SM5-1 (4OSN and 4OT1) (left) and domain II of gB, chain B, modeled with Fab SM5-1 (right) to illustrate how glycosylation would affect antibody binding. The heavy chain of SM5-1 is colored in magenta, the light chain is in slate, and AD-4 epitope is in lime. (D) A close-up view of domain I (AD-5). Residue involved in binding of 1G2 (N284) is colored in magenta, residues involved in binding of 2C2 (N293/D295) are in blue, and residue important for binding of both (Y280) is in purple. Residues shown not to affect binding of 1G2 or 2C2 are colored in pale blue.

Antigenic site AD-1, located within DIV, produces the strongest immune response [21, 24, 48–50]. DIV has only two N-linked glycosylation sites, and most of its surface is exposed and available for antibody binding (Fig 6A and 6B), which likely accounts for its high immunogenicity. To understand the structural determinants for neutralizing ability of some anti-AD-1 antibodies, we mapped the locations of residues shown to be important for antibody binding, which were identified in a study that characterized the effect of 600 random mutations, generated in a construct encompassing DIV (residues 484–650), on binding of a panel of anti-AD-1 antibodies [51]. Fifteen mutations (R562C, P577L, S587L, Y588C, G592S, G595D, L601P/H605N, C610Y, L612F, P613Y, Y625C, Y627C, F632L, and K633T) reduced or abolished binding of one or more tested antibodies, seven neutralizing and four non-neutralizing [51]. Several of these residues are buried and inaccessible by antibodies while the rest are surface-exposed and could be directly involved in antibody binding (Fig 6B). Mutations P577L, C610Y, Y627C, and K633T reduced binding of all tested antibodies. P577 and Y627 are located next to each other within the DIV core while C610 participates in a conserved disulfide bond. Thus, all three residues likely help maintain the structural integrity of DIV and, therefore, the entire antigenic site AD-1. By contrast, K633 is fully exposed, so it is unclear how K633T mutation would perturb binding of all tested antibodies. Each of the other mutations affected binding of a subset of antibodies, which suggests that AD-1 contains multiple overlapping epitopes. Unfortunately, the structure does not explain why some antibodies that bind AD-1 are neutralizing while others are not. Most mutations reduce binding of a subset of antibodies, which includes both neutralizing and non-neutralizing ones, making it challenging to explain or predict the neutralization capacity. Nevertheless, two mutations, F632L and G595D, specifically reduced binding of several (although not all) neutralizing antibodies without affecting the binding of non-neutralizing antibodies. These residues are located on the opposite sides of DIV: G595 is buried at the DIV/DIII interface, and a conformational change would be required to expose it. F632 is partially exposed on the surface. It is tempting to speculate that neutralizing antibodies bind the epitopes exposed in the prefusion state whereas non-neutralizing antibodies probably bind the epitopes that are only accessible in the postfusion state. If so, we would expect both F632 and G595 to be exposed on the surface of the prefusion form of gB. The discernment of the structural basis of neutralization awaits the structures of gB bound to neutralizing vs. non-neutralizing anti-AD-1 antibodies.

The two subdomains of antigenic site AD-2, site I (residues 68–77) and site II (residues 50–54), are located within the flexible N-terminus of gB that is absent from the crystallized construct. The sequence of site II is highly variable in clinical strains, while site I is highly conserved and is likely functionally important, which helps explain why antibodies targeting it are neutralizing [25]. Unlike the unglycosylated site II, site I contains 2 conserved predicted glycosylation sites within 10 residues (S4 Fig). Glycans could reduce antibody binding resulting in the observed mild immunogenicity [21, 25].

Antigenic site AD-4 is located within DII (Fig 6A and 6C) and was defined by the epitope of human neutralizing mAb SM5-1 isolated from B cells of a seropositive patient [21]. The recently published structure of an isolated HCMV gB DII bound to SM5-1 Fab [52] revealed how the antibody binds the narrow epitope at a sharp angle (Fig 6C). But, DII in the crystallized complex was expressed in *E*. *coli* and lacks glycosylation. The glycan at position N383, located next to the SM5-binding site, is incompatible with SM5-1 binding in the orientation observed in the unglycosylated DII/SM5-1 complex due to a potential clash with the antibody loop CDR H3 (Fig 6C). We hypothesize that in the fully glycosylated gB, the SM5 antibody may bind at an even sharper angle to avoid steric hindrance due to the N383 glycan, which could reduce the surface area of its binding site and thus the affinity.

Antigenic site AD-5 is located within DI and contains two distinct epitopes defined by human mAbs 1G2 and 2C2, isolated from two seropositive individuals (Fig 6A and 6D) [53]. Residues important for the ability of 1G2 or 2C2 to neutralize virus in cell culture, Y280/N284 and Y280/N293/D295, respectively, were identified by mutagenesis [53]. Mutation Y280A reduced neutralization by both antibodies, which suggested that Y280 could be directly involved in antibody binding [53]. In the structure, Y280 is almost completely buried, however, and we conclude that instead of interacting with the antibodies directly, Y280 instead buttresses the surface region that is directly involved in binding. All residues involved in binding of anti-AD-5 neutralizing antibodies are conserved amongst the analyzed strains (S4 Fig), supporting the idea that this region is functionally important. The residues that define the binding sites of 1G2 and 2C2 are surrounded by glycans, at position N208, N281, N286, N302, which could hinder antibody binding. Neutralization by 1G2 and 2C2 in cell culture increased ~3-fold when N286 was mutated to alanine, despite no obvious increase in antibody binding to gB [53]. The glycan shield could counteract the high neutralizing potency of anti-AD-5 antibodies, which could explain why such antibodies are less common. Further mutagenesis and, ultimately, the structures of gB bound to 1G2 or 2C2 would be necessary to pinpoint additional residues involved in binding and help explain how these antibodies avoid glycans.

## Discussion

The 3.6-Å crystal structure of the HCMV gB ectodomain reported here is the first structure of any glycoprotein from a betaherpesvirus. HCMV gB structure resembles the postfusion structures of HSV-1 and EBV homologs, making it a member of the new class III viral fusogens. Despite structural similarities, each gB has a unique domain arrangement, demonstrating structural plasticity of gB that may serve to accommodate virus-specific functional requirements. By contrast, the postfusion forms of G homologs from Vesicular Stomatitis Virus [18] and Chandipura virus [54], which also belong to class III viral fusogens, have very similar structures, including domain orientations. These observations suggest different constraints on class III fusogens from different viral families, which may reflect differences either in their mode of activation (pH for vesiculovirus G vs. interaction with additional viral glycoproteins for herpesvirus gB), or in the architecture and the stabilization of their pre-fusion conformations, or both.

The structure illustrates how most of the gB surface is shielded by a thick glycan layer. Viruses commonly use glycosylation to escape immune recognition (reviewed in [55]). Glycosylation alteration in HIV-1 Env has been proposed as a mechanism of immune escape [56]. High conservation of the glycosylation pattern in HCMV gB suggests that extensive glycosylation in HCMV gB may instead protect functionally important regions from immune recognition. DI and DII are the most heavily glycosylated, and all antibodies against the antigenic sites located in these domains are neutralizing. Although the functional roles of domains DI and DII in HCMV gB have not yet been elucidated, these domains have been proposed to bind gH/gL in HSV-1 gB [57], and in HCMV gB could, perhaps, interact with the gH/gL pentamer or the trimer. Carbohydrates may be shielding antigenic sites within these domains from immune recognition as a means of avoiding neutralization, which could explain why such antibodies against AD-4 and AD-5 are relatively rare compared to the number generated against AD-1.

The core of the gB trimer, consisting of DIII and DV, is not glycosylated. These regions are expected to undergo large-scale refolding during the prefusion-to-postfusion transition [34]. Fusion subunits of other viral fusogens such as HIV Env [45] and Ebola gp [46] are similarly shielded by heavily glycosylated domains in the prefusion conformation, hinting at a common strategy for immune evasion. No antibodies against DIII or DV have yet been isolated, and we expect that DIII and DV in the prefusion form of gB to be protected from the immune response by the glycan shields of DI and DII.

Although HCMV gB elicits a strong immune response in humans and induces the production of neutralizing antibodies, most anti-gB Abs are non-neutralizing and target the immunodominant antigenic site AD-1 [21, 22]. The limited glycosylation of DIV, containing AD-1, could account for this immunodominance (Fig 6B). Structure analysis is consistent with the presence of multiple overlapping epitopes within AD-1 but does not explain why most antibodies that bind AD-1 are non-neutralizing. One plausible explanation is that neutralizing antibodies bind the epitopes exposed in the prefusion state whereas non-neutralizing antibodies probably bind the epitopes that are only accessible in the postfusion state. The HSV-1 envelope may display both the prefusion and the postfusion form of gB [58, 59], which raises the possibility that the postfusion form of gB could be present on the surface of HCMV. By presenting the postfusion form with its fully exposed AD-1, HCMV could divert the immune response towards production of non-neutralizing antibodies.

In summary, the crystal structure of HCMV gB ectodomain provides an important framework for elucidating the immunogenic determinants and establishes HCMV gB as the viral fusogen. The glycan distribution in HCMV gB revealed by its structure suggests that antigenic sites that elicit neutralizing antibodies are more heavily glycosylated than those that elicit non-neutralizing antibodies. By using glycans to shield neutralizing epitopes while exposing regions that elicit non-neutralizing antibodies, gB could be redirecting the immune response.

## Materials and Methods

### Cloning and mutagenesis

Previously, the ectodomain of gB (strain AD169), residues 25–706 (gB706), was cloned into pFastBac1 plasmid for insect cell expression using PCR with primers and 4 hydrophobic residues in the predicted fusion loops were mutated to their non-hydrophobic HSV-1 and EBV counterparts (Y155G/I156H/Y157R/W240A) [27]. Three additional residues were also mutated (Y206H/L241T/Y242H) using splicing by overlap extension (SOE) PCR with primers (forward flanking: 5'-CGGTCTAGAACCATGAAATTCTT-3', Y206H mutation: forward 5’-CATAGGGACAGTCATGAAAACAAAACC-3’; reverse 5’-GGTTTTGTTTTCATGACTGTCCCTATG-3’; L241T and Y242H mutations: forward 5’-GGCAGCACCGCGACCCATCGT-3’; reverse 5’-ACGATGGGTCGCGGTGCTGCC-3’, reverse flanking: 5’-CGCGCATATGTTTGATTGTAT-3’) and cloning into pFastBac1::gB706-4M plasmid (pSS2) using the restriction enzymes XbaI and NdeI, generating the construct pFastBac1::gB706-7M (pSS9).

The N-terminus of gB706-7M was truncated prior to amino acid 78 (gB78-706-7M) using primers (forward flanking 5’-TACTACGGAGCAAGTTCCCGA-3’; Δ5’ reverse 5’-ACTCCCACCACATCTCCGTACGCTAGCGCATAGATGTAAGAAATG-3’; Δ3’ forward 5’-CATTTCTTACATCTATGCGCTAGCGTACGGAGATGTGGTGGGAGT-3’; reverse flanking 5’-TGGTTTCGAAGACGGACACGTT-3’). Cloning was accomplished using plasmid restriction site SacI and endogenous restriction site NdeI, generating construct pFastBac1::gB78-706-7M (pHB14). The endogenous furin cleavage site was replaced with an enterokinase cleavage site (gB78-706-7M-E) using primers (forward flanking 5’-ATCGCAATGCCAGCTACTTTG-3’; enterokinase 5’ reverse 5’-CTTATCATCATCATCATGAGTGATATTCAGACTGGATC-3’; enterokinase 3’ forward 5’-GATGATGATGATAAGAGTACGAGTGACAATAATACAACT-3’; reverse flanking 5’- CTACAAATGTGGTATGGCTGATT-3’) containing an enterokinase cleavage site (DDDDK) (underlined), generating construct pFastBac1::gB78-706-7M-E (pHB15). Endogenous internal NdeI and HindIII restriction sites were utilized for cloning into gB78-706-7M. All clones were sequenced and verified to contain the correct reading frame and appropriate sequences. Due to the cloning strategy, all mature proteins contain two extra residues (DP) at the N terminus.

### Antibodies

Hybridoma cell line expressing anti-HCMV gB monoclonal antibody 27–39 was a gift from William J. Britt (University of Alabama). The monoclonal antibodies were purified at the GRASP facility at Tufts Medical Center. 27–39 is a conformational mAb that recognizes the oligomeric form of HCMV gB ectodomain [60].

### Viruses and cells

Spodoptera frugiperda (Sf9) cells were grown in SF-900 II SFM (Invitrogen) in suspension at 27°C. Recombinant baculoviruses of all HCMV gB ectodomain constructs were generated using Bac-to-Bac system (Invitrogen). After two rounds of amplification, passage 3 (P3) stocks of baculoviruses were harvested and stored at 4°C in the dark and in the presence of 2% Fetal Bovine Serum (FBS, Invitrogen).

### Protein expression and purification

Purification of HCMV gB ectodomain from supernatants of Sf9 cells infected with recombinant baculovirus has been previously described [27]. Briefly, ~7.5 mL of viral stocks were added to 1.5 L of SF9 cells at 2x10^6^ cells/mL. Supernatant was harvested at 68–72 hours post infection by pelleting cells at 3750 rpm at 4°C for 1 hour. The supernatant was filtered (0.45 μm filter), then concentrated by Tangential Flow Filtration with a 20-KDa PLTK cartridge (Millipore). To decrease media components, the buffer was exchanged once with phosphate-buffered saline and 0.1 mM PMSF was added as a protease inhibitor. gB was purified from the concentrated supernatant via immunoaffinity chromatography, with mAb 27–39 coupled to CNBr-activated Sepharose 4B (GE Healthcare). The column was washed with 10 mM tris (pH 8.0), 500 mM NaCl and gB was eluted with 3 M KSCN in wash buffer.

### N-terminal sequencing

For N-terminal sequencing, protein samples were resolved by a 4–15% SDS-PAGE and transferred to a PVDF membrane. The membrane was stained with Coomassie R-250. The protein bands of interest were cut out and submitted for sequencing by Edman degradation at the Tufts University Core Facility.

### Crystallization

Purified gB706-7M failed to crystallize, but both trypsin-cleaved gB706-7M and gB78-706-7M formed crystals in the presence of PEG 8000 as precipitant. Crystals were grown by vapor diffusion in hanging drops using 1 μL protein at ~5 mg/ml and 1 μL crystallization solution (10–14% PEG 8000, 0.1 M NaCl, 0.15 M Mg(NO_3_)_2_) at room temperature and flash frozen in solution identical to the well solution plus 20% glycerol for data collection. X-ray diffraction data were collected at 100 K on NE-CAT beamlines 24IDC and 24IDE at the Advanced Photon Source, Argonne National Laboratory, and processed using XDS [61] as implemented in RAPD (https://rapd.nec.aps.anl.gov/rapd). All crystals took orthorhombic space group and had cell dimensions consistent with 3 gB molecules (one trimer) per asymmetric unit. Data were processed in P222 space group. Many crystals had to be tested to identify those that diffracted to medium resolution, 3.6-Å, but these crystals were prone to radiation damage, and the collected data sets had low completeness. The crystals of uncleaved gB78-706-7M-E diffracted better on average and provided a complete 3.6-Å data set used for structure determination.

### Structure determination

Molecular replacement as implemented in *Phaser-MR* [62] yielded a clear solution with correct packing only when using the P2_1_2_1_2_1_ space group and the structure of trimeric EBV gB ectodomain (PDB ID 3FVC) [17] as a search model. The structure of trimeric HSV-1 gB ectodomain (PDB ID 2GUM) [16] did not yield a clear solution. However, the resulting maps were streaky, likely due to low data completeness. The first interpretable maps were obtained when molecular replacement was carried out with gB78-706-7M-E dataset and the EBV gB ectodomain as a search model. Density modification including 3-fold averaging, solvent flattening, and histogram matching, as implemented in *Autosol* [62] resulted in good-quality maps. *Autobuild* [62] was used to trace ~30% of the model; the rest was built manually in *Coot* [63] using density-modified maps generated by *Autobuild*. Domain II of HCMV gB (PDB ID 4OT1) [52] and domains I and IV of HSV-1 gB (PDB ID 2GUM) [16] were manually positioned into the density and rebuilt in *Coot*. Extensive rebuilding was necessary because neither HSV-1 nor EBV structures fit well into the experimental density. Prior to refinement, 5% of reflections set aside as a reference. The model was refined using gradient minimization and thermal parameter refinement as implemented in *phenix*.*refine* [62]. NCS and secondary structure were restrained initially. Several rounds of alternating refinement and rebuilding decreased R to 29.0% and R_free_ to 31.8%. At this point, secondary structure restraints were released, and the model underwent several additional rounds of refinement and rebuilding. The final R_work_ is 24.82% and R_free_ is 27.45%. Relevant crystallographic statistics are in Table 1. The final model has residues 87–696 in chain A (unresolved 78–86, 117–120, 409–410, 435–475), residues 86–697 in chain B (unresolved 78–85, 116–121, 439–474), and residues 83–695 in chain C (unresolved 78–82, 117–118, 441–475), one calcium ion located at the three-fold symmetry axis, and two water molecules.

**Table 1:** Data collection and refinement statistics.

	gB78-706-7M-E
***Data collection*** ^***a***^	
Space group	P2_1_2_1_2_1_
Cell dimensions	
*a*, *b*, *c* (Å)	92.15, 134.10, 295.56
α, β, γ (°)	90, 90, 90
Resolution (Å)	147.7–3.6 (3.75–3.6)
*R*_sym_ or *R*_merge_	0.167 (0.961)
*I*/σ*I*	9.0 (1.6)
Completeness (%)	99.4 (99.9)
Redundancy	4.1 (4.1)
Wilson B	88.37
***Refinement***	
Resolution (Å)	57.63–3.60
No. reflections (free)	42515 (1891)
*R*_work_/*R*_free_ ^b^	0.2482/0.2745
No. atoms	14380
Protein	14377
Ca	1
Solvent	2
*B*-factors	115.5
Protein	115.5
Ca	85.7
Solvent	83.1
RMS^c^ deviations	
Bond lengths (Å)	0.005
Bond angles (°)	1.024
Ramachandran plot^d^	
Favored (%)	97.04
Allowed (%)	2.78
Outliers (%)	0.18
All-atom clash score^d^	12.81

^a^Values in parentheses are for highest-resolution shell.

^b^R_work_ and R_free_ are defined as ∑||F_obs_|-|F_calc_||/∑|F_obs_| for the reflections in the working or the test set, respectively.

^c^RMS, root mean square.

^d^As determined using Molprobity (molprobity.biochem.duke.edu) [64].

## Supporting Information

S1 Fig Mutations in the putative fusion loops. Mutated residues in the putative fusion loops are shown using a HCMV gB homology model generated using the structure of HSV-1 gB. Residues are colored as follows: hydrophobic (yellow), positively charged (blue), negatively charged (red), uncharged (grey), and histidines (cyan). Side chains of mutated residues in HCMV gB706 and gB706-7M mutant are labeled (in only one protomer, for simplicity). A sequence alignment of the HCMV gB706, HCMV gB706-7M, HSV-1 gB and EBV gB fusion loops shows which mutations were introduced to generate the gB706-7M mutant (color scheme maintained).
(TIF)

S2 Fig Size-exclusion chromatography of HSV-1 gB730, HCMV gB706, and HCMV gB706-7M. Size exclusion chromatograms of HSV-1 gB ectodomain (gB730, green), HCMV gB ectodomain (gB706, red), and HCMV gB ectodomain with the putative 7 hydrophobic fusion loop residues mutated (gB706-7M, purple) are overlaid. Elution volumes of the size-exclusion standards and the void volume are labeled with arrows.
(TIF)

S3 Fig Gels of various HCMV gB constructs. gB78-706-7M (partial cleavage during expression), trypsin cleaved gB78-706-7M (non-specific cleavage), furin cleaved gB78-706-7M (incomplete cleavage), and gB78-706-7M-E (uncleaved) constructs were expressed and purified in SF9 cells and analyzed by SDS-PAGE and Coomassie staining. Arrows indicated uncleaved gB monomers and the cleavage products, ~70 kDa N terminus and ~35 kDa C terminus.
(TIF)

S4 Fig Sequence alignment of gB from clinical and laboratory-adapted HCMV strains. Sixty HCMV gB sequences from clinical and laboratory-adapted strains, downloaded from NCBI’s RefSeq data base, were aligned and analyzed using ClustalW2 [32] and ESPript 3.x [33]. Identical residues are shown as white text on red background and similar residues are highlighted in yellow.
(PDF)

S5 Fig Mapping of poorly conserved residues within HCMV gB. Surface representation of gB with non-conserved residues displayed in red, semi-conserved residues in orange, conserved residues in yellow, and completely conserved residues in grey. Glycans (teal) are shown in space-filled representation. Side and top views are shown.
(TIF)

S6 Fig Alignment of individual gB domains. Individually aligned domains IV, III, and II of HSV-1 (2GUM) (green), HCMV (orange), and EBV (3FVC) (cyan) are shown side by side and as an overlay. Residues used in alignments and RMSDs are listed in S1 Table. Boxes indicate the location of the aligned region within the HCMV gB structure.
(TIF)

S7 Fig Putative disintegrin-like-domain (DLD) is largely buried. Residues in the putative DLD motif required for integrin binding, R92 (red), D101 (blue), L102 (green) are highlighted on the structure of HCMV gB. F105 is completely buried.
(TIF)

S1 Table Residues used in domain alignments and RMSDs of HSV-1, HCMV, and EBV gB. Residues used in aligning individual domains of the three homologues are listed. RMSDs between HSV-1 and HCMV gB domains are listed under HSV-1. RMSDs between EBV and HCMV gB domains are listed under EBV. Pymol (http://www.pymol.org) was used to calculate RMSDs.
(PDF)
