# Correction: Crystal Structure of the Human Cytomegalovirus Glycoprotein B

**DOI:** 10.1371/journal.ppat.1005300

**Published:** 2015-11-17

**Authors:** Heidi G. Burke, Ekaterina E. Heldwein

The Funding Statement is missing funding information from the NIH. Please see the full Funding Statement here.

This study was funded by the Burroughs Wellcome Fund Award for Investigators in Pathogenesis (EEH) and the NIH T32-AI007422 (HGB). This work is based upon research conducted at the Northeastern Collaborative Access Team beamlines, which are funded by the National Institute of General Medical Sciences from the National Institutes of Health (P41 GM103403). The funders had no role in study design, data collection and analysis, decision to publish, or preparation of the manuscript.
