# Supplementary figures and images for: Crystal Structure of the Human Cytomegalovirus Glycoprotein B
Source: PLoS Pathog. 2015 Oct 20;11(10):e1005227. doi: 10.1371/journal.ppat.1005227 (PMC4617298; doi:10.1371/journal.ppat.1005227)

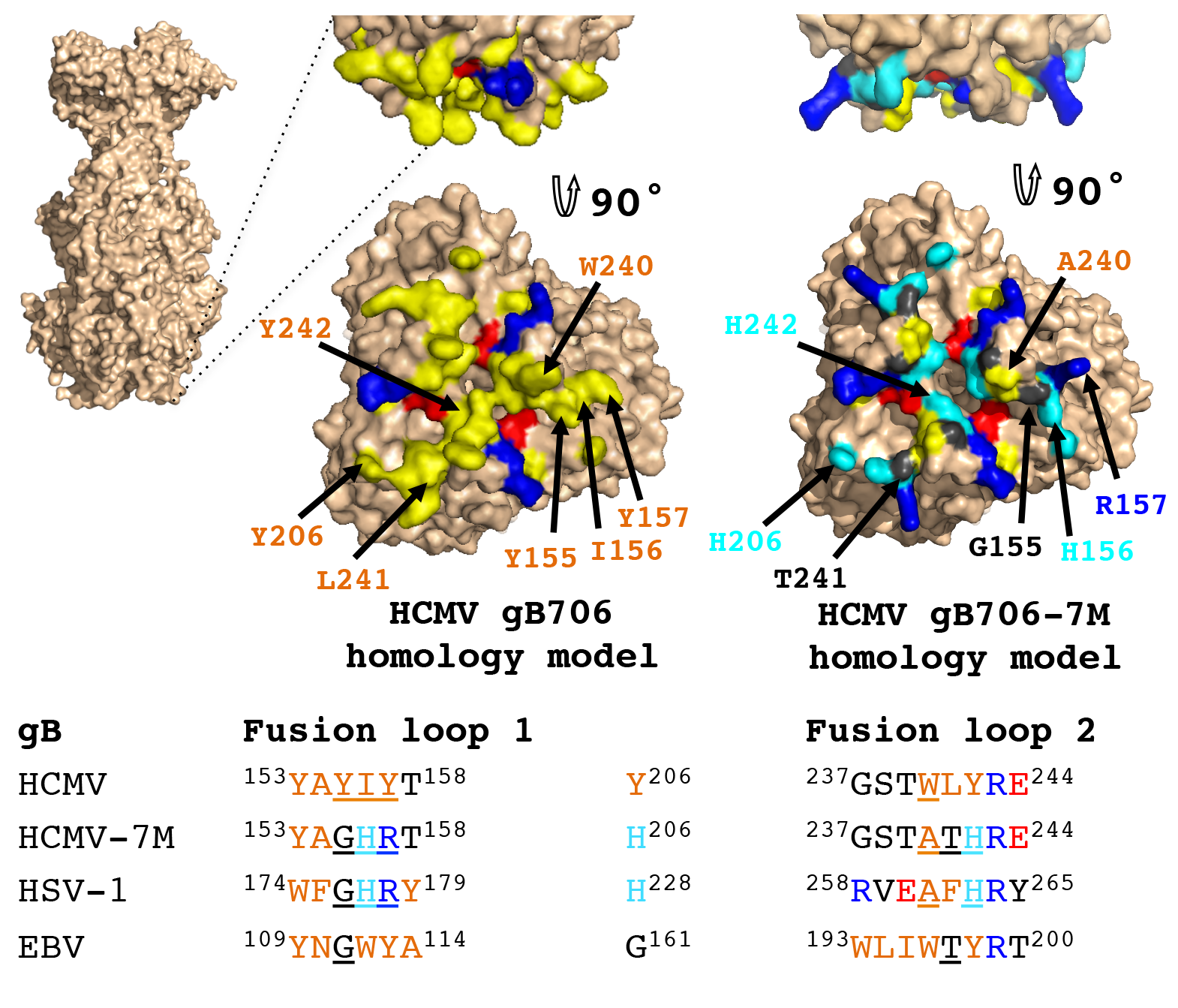

Supplement: S1 Fig — Mutated residues in the putative fusion loops are shown using a HCMV gB homology model generated using the structure of HSV-1 gB. Residues are colored as follows: hydrophobic (yellow), positively charged (blue), negatively charged (red), uncharged (grey), and histidines (cyan). Side chains of mutated residues in HCMV gB706 and gB706-7M mutant are labeled (in only one protomer, for simplicity). A sequence alignment of the HCMV gB706, HCMV gB706-7M, HSV-1 gB and EBV gB fusion loops shows which mutations were introduced to generate the gB706-7M mutant (color scheme maintained). (TIF) [file ppat.1005227.s001.tif]

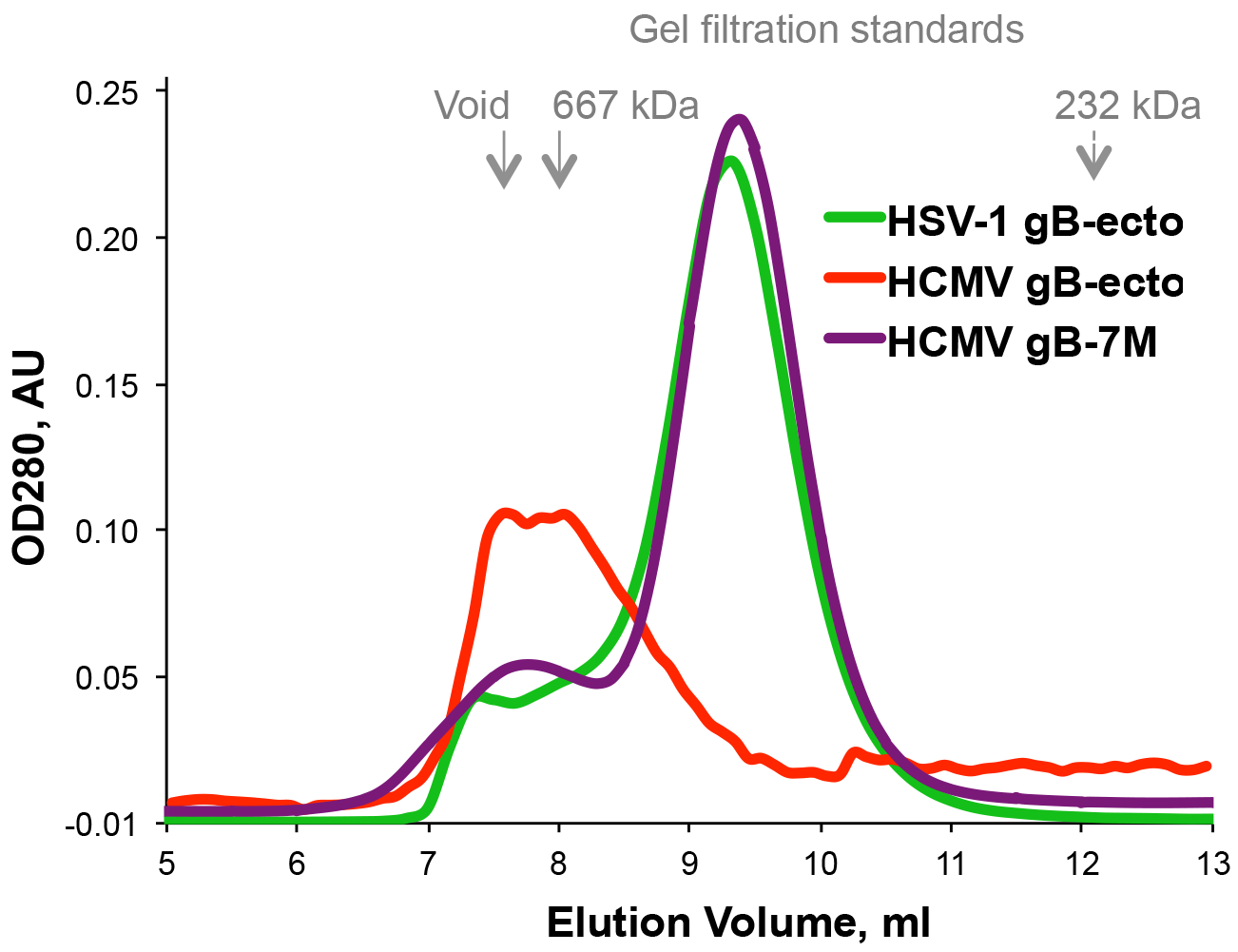

Supplement: S2 Fig — Size exclusion chromatograms of HSV-1 gB ectodomain (gB730, green), HCMV gB ectodomain (gB706, red), and HCMV gB ectodomain with the putative 7 hydrophobic fusion loop residues mutated (gB706-7M, purple) are overlaid. Elution volumes of the size-exclusion standards and the void volume are labeled with arrows. (TIF) [file ppat.1005227.s002.tif]

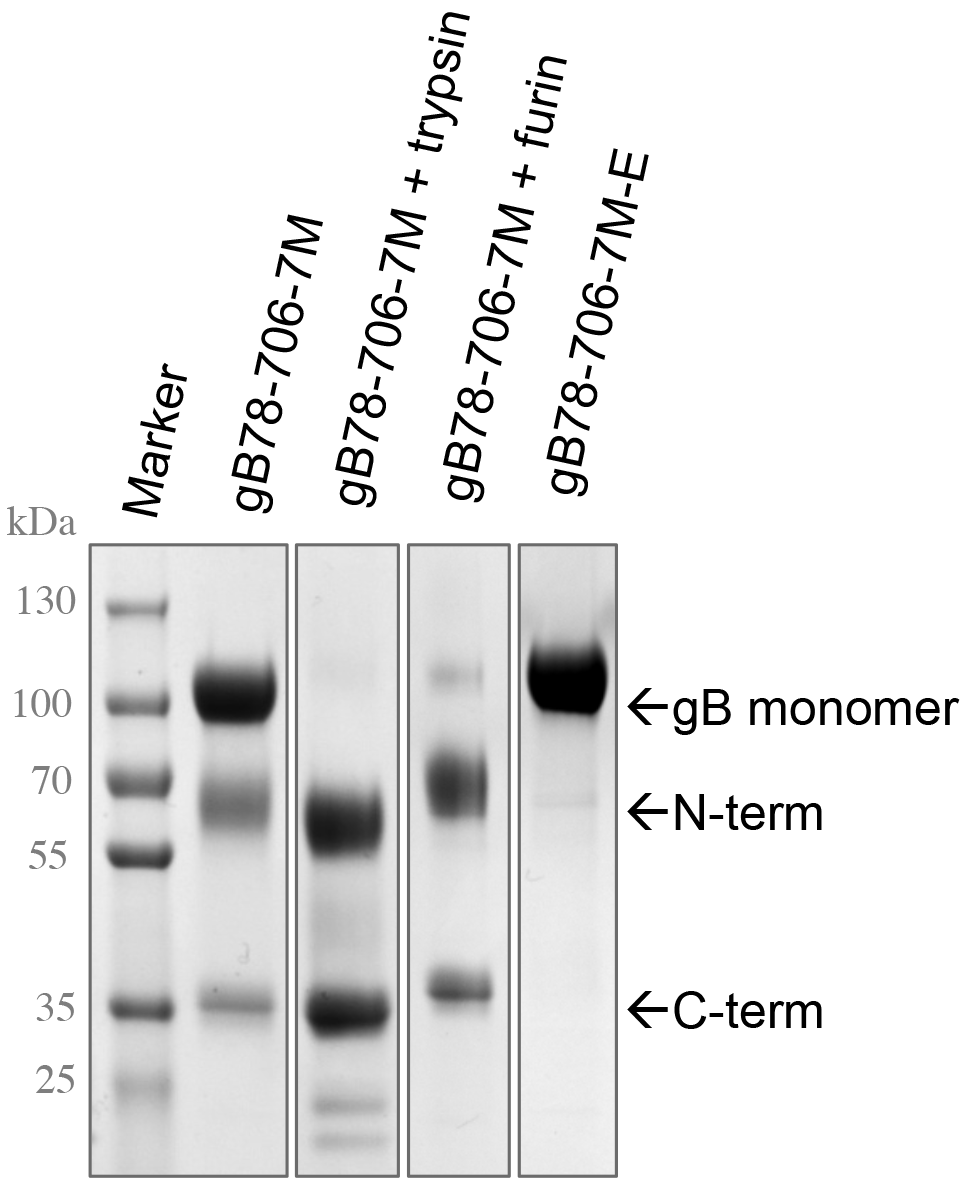

Supplement: S3 Fig — gB78-706-7M (partial cleavage during expression), trypsin cleaved gB78-706-7M (non-specific cleavage), furin cleaved gB78-706-7M (incomplete cleavage), and gB78-706-7M-E (uncleaved) constructs were expressed and purified in SF9 cells and analyzed by SDS-PAGE and Coomassie staining. Arrows indicated uncleaved gB monomers and the cleavage products, ~70 kDa N terminus and ~35 kDa C terminus. (TIF) [file ppat.1005227.s003.tif]

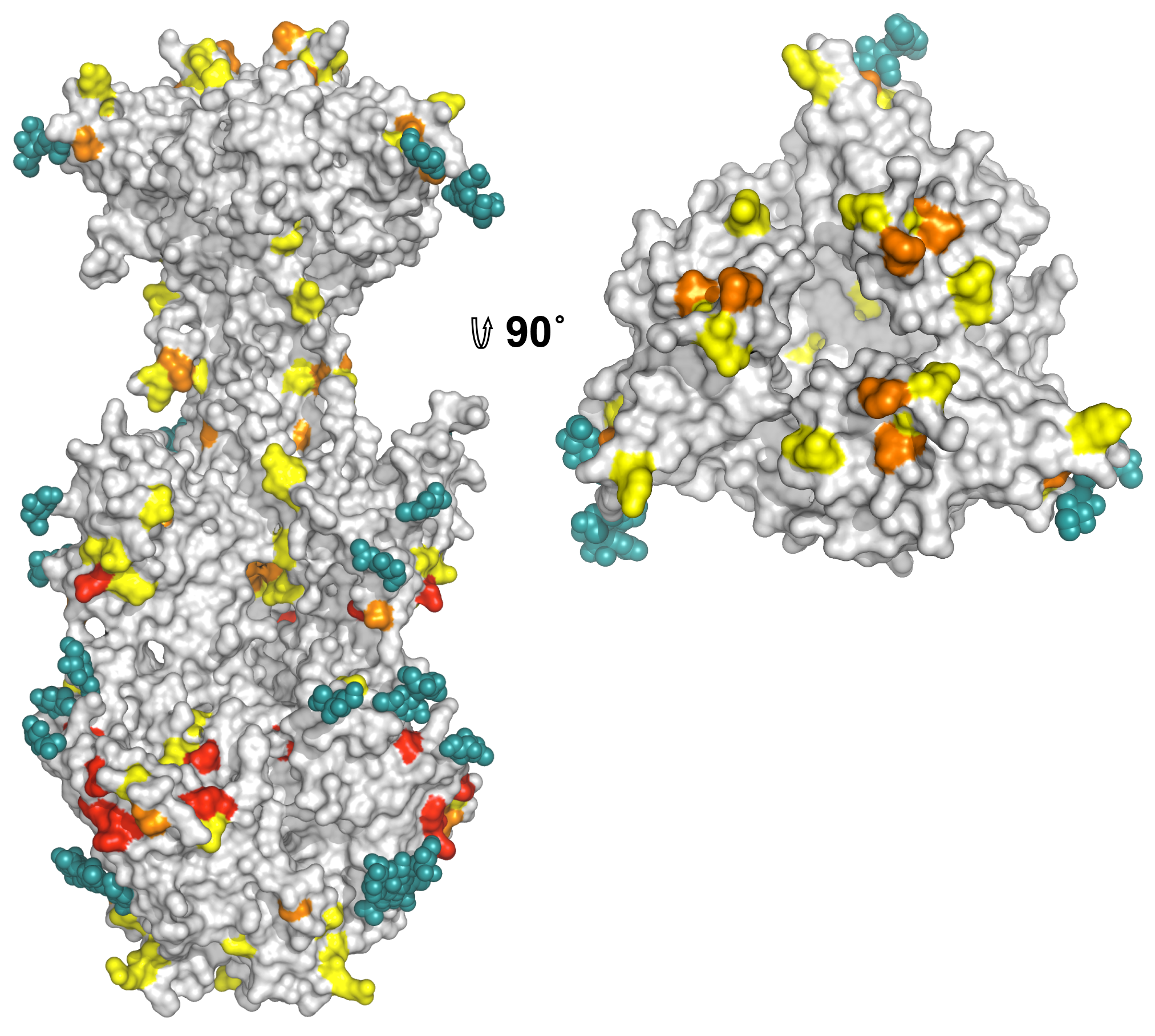

Supplement: S5 Fig — Surface representation of gB with non-conserved residues displayed in red, semi-conserved residues in orange, conserved residues in yellow, and completely conserved residues in grey. Glycans (teal) are shown in space-filled representation. Side and top views are shown. (TIF) [file ppat.1005227.s005.tif]

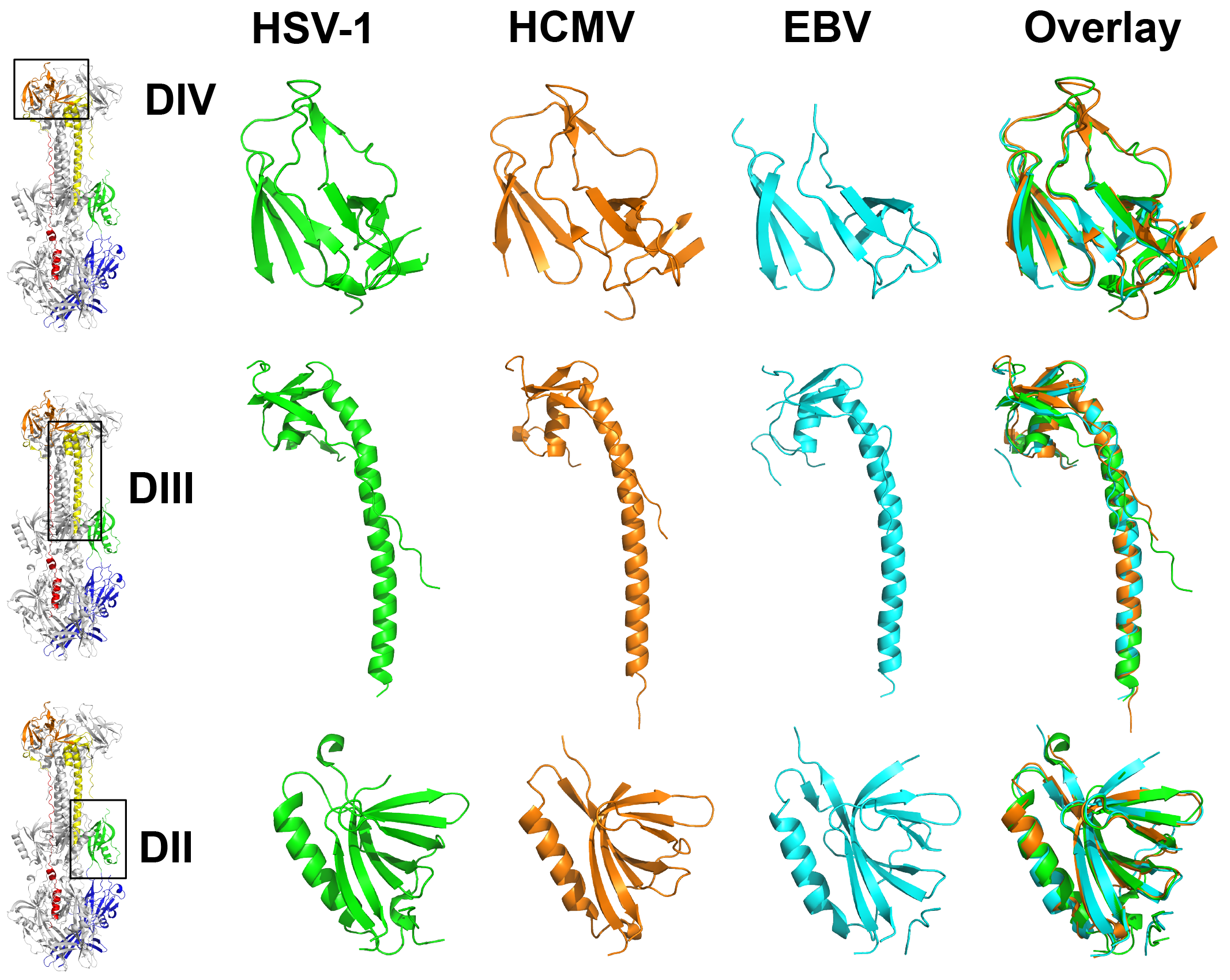

Supplement: S6 Fig — Individually aligned domains IV, III, and II of HSV-1 (2GUM) (green), HCMV (orange), and EBV (3FVC) (cyan) are shown side by side and as an overlay. Residues used in alignments and RMSDs are listed in S1 Table. Boxes indicate the location of the aligned region within the HCMV gB structure. (TIF) [file ppat.1005227.s006.tif]

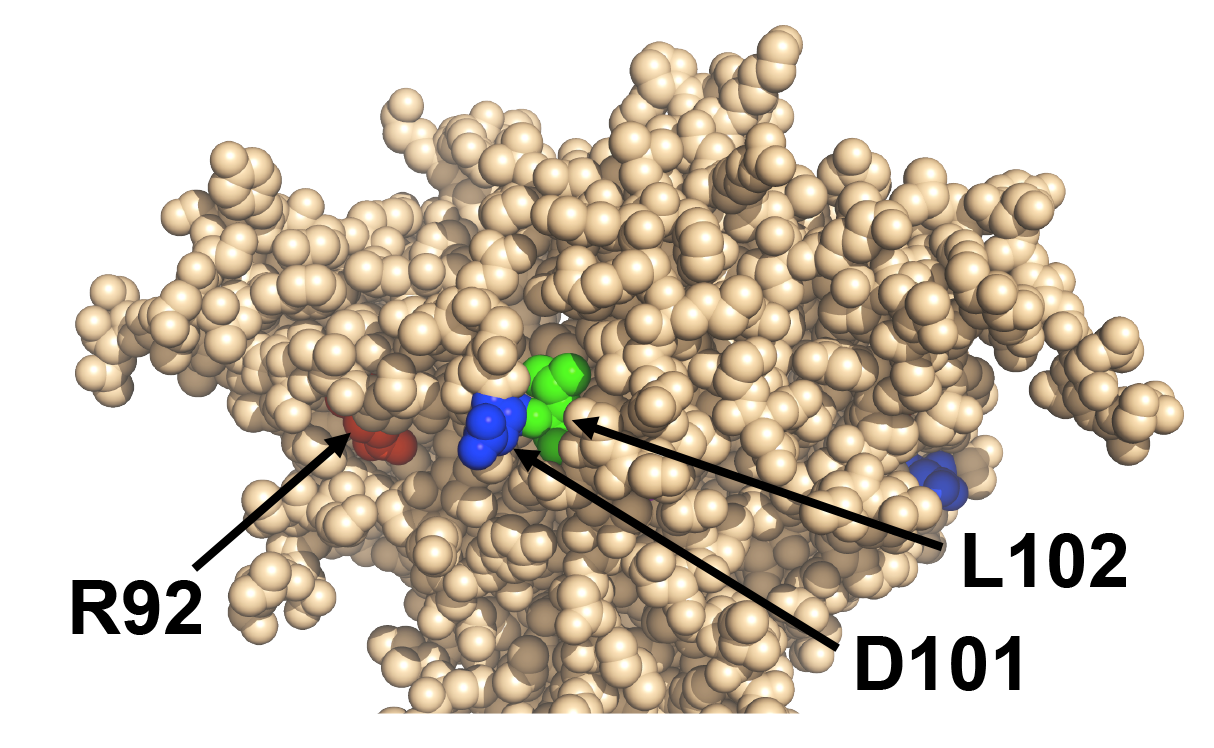

Supplement: S7 Fig — Residues in the putative DLD motif required for integrin binding, R92 (red), D101 (blue), L102 (green) are highlighted on the structure of HCMV gB. F105 is completely buried. (TIF) [file ppat.1005227.s007.tif]
