# Supplementary material for: Crystal Structure of the Human Cytomegalovirus Glycoprotein B
Source: PLoS Pathog. 2015 Oct 20;11(10):e1005227. doi: 10.1371/journal.ppat.1005227 (PMC4617298; doi:10.1371/journal.ppat.1005227)
Supplement: S4 Fig — Sixty HCMV gB sequences from clinical and laboratory-adapted strains, downloaded from NCBI’s RefSeq data base, were aligned and analyzed using ClustalW2 [32] and ESPript 3.x [33]. Identical residues are shown as white text on red background and similar residues are highlighted in yellow. (PDF) [file ppat.1005227.s004.pdf]

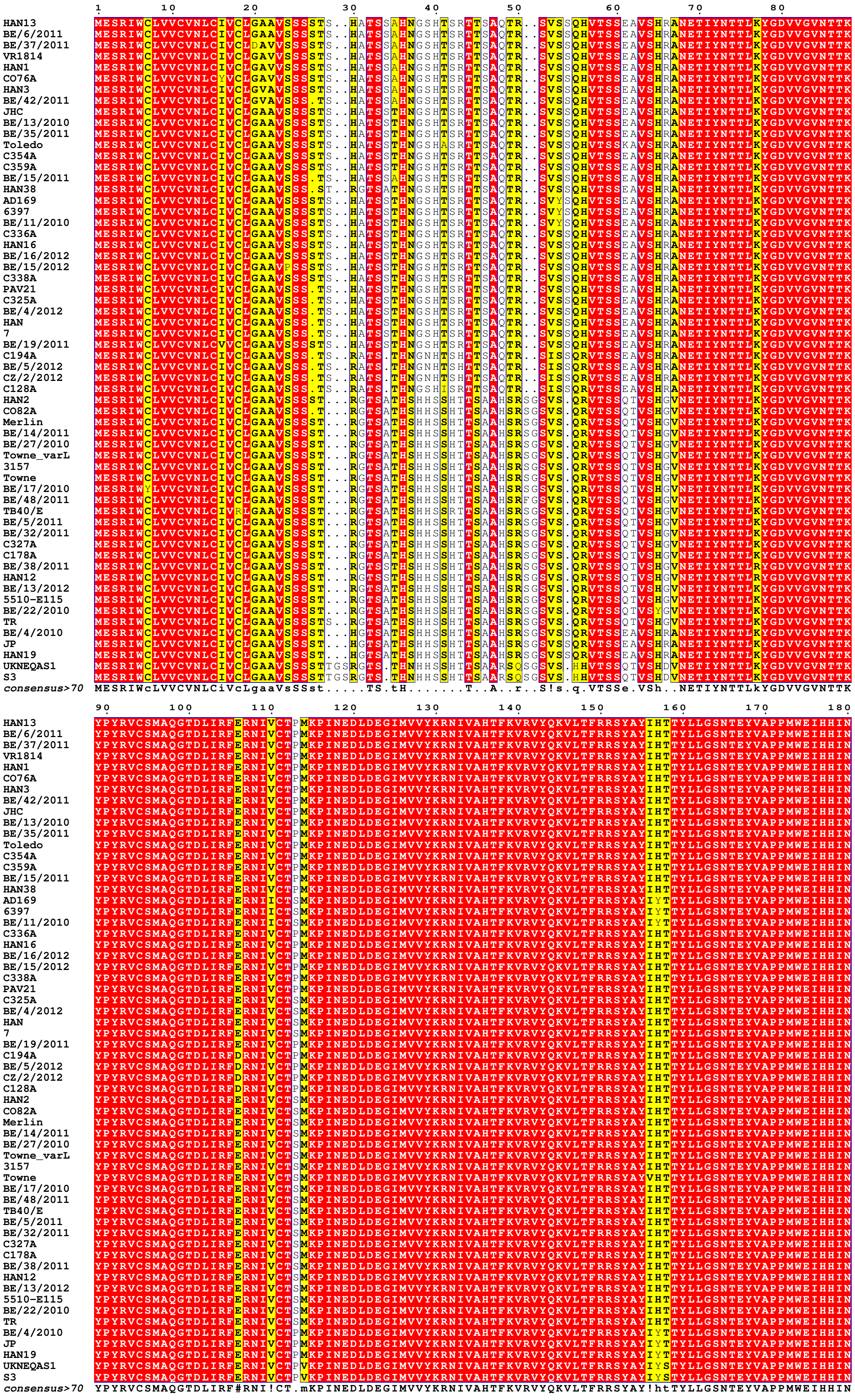
1 10 20 30 40 50 60 70 80
HAN13
BE/6/2011
BE/37/2011
VR1814
HAN1
CO76A
HAN3
BE/42/2011
JHC
BE/13/2010
BE/35/2011
Toledo
C354A
C359A
BE/15/2011
HAN38
AD169
6397
BE/11/2010
C336A
HAN16
BE/16/2012
BE/15/2012
C338A
PAV21
C325A
BE/4/2012
HAN
7
BE/19/2011
C194A
BE/5/2012
CZ/2/2012
C128A
HAN2
CO82A
Merlin
BE/14/2011
BE/27/2010
Towne_varL
3157
Towne
BE/17/2010
BE/48/2011
TB40/E
BE/5/2011
BE/32/2011
C327A
C178A
BE/38/2011
HAN12
BE/13/2012
5510-E115
BE/22/2010
TR
BE/4/2010
JP
HAN19
UKNEQAS1
S3
consensus>70 MESRIWcLVVCVNLCiVcLgaaVsSSst...TS.tH.......T..A..r..S!s.q.VTSSe.VSh..NETIYNTTLkYGDVVGVNTTK
90 100 110 120 130 140 150 160 170 180
consensus>70 YPYRVCSMAQGTDLIRF#RNI!CT.mKPINEDLDEGIMVVYKRNIVAHTFKVRVYQKVLTFRRSYAY!htTYLLGSNTEYVAPPMWEIHHIN

```
                  190         200         210         220         230         240         250         260         270
HAN13          RHSQCYSSYSRVIAGTVFVAYHRDSYENKTMQLMLDDYSNTHSTRYVTVKDQWHSRGSTWLYRETCNLNCMVTITTARSKYPYHFFATSTGD
BE/6/2011      RHSQCYSSYSRVIAGTVFVAYHRDSYENKTMQLMLDDYSNTHSTRYVTVKDQWHSRGSTWLYRETCNLNCMVTITTARSKYPYHFFATSTGD
BE/37/2011     RHSQCYSSYSRVIAGTVFVAYHRDSYENKTMQLMLDDYSNTHSTRYVTVKDQWHSRGSTWLYRETCNLNCMVTITTARSKYPYHFFATSTGD
VR1814         RHSQCYSSYSRVIAGTVFVAYHRDSYENKTMQLMLDDYSNTHSTRYVTVKDQWHSRGSTWLYRETCNLNCMVTITTARSKYPYHFFATSTGD
HAN1           RHSQCYSSYSRVIAGTVFVAYHRDSYENKTMQLMLDDYSNTHSTRYVTVKDQWHSRGSTWLYRETCNLNCMVTITTARSKYPYHFFATSTGD
CO76A          RHSQCYSSYSRVIAGTVFVAYHRDSYENKTMQLMLDDYSNTHSTRYVTVKDQWHSRGSTWLYRETCNLNCMVTITTARSKYPYHFFATSTGD
HAN3           RHSQCYSSYSRVIAGTVFVAYHRDSYENKTMQLMLDDYSNTHSTRYVTVKDQWHSRGSTWLYRETCNLNCMVTITTARSKYPYHFFATSTGD
BE/42/2011     RHSQCYSSYSRVIAGTVFVAYHRDSYENKTMQLMLDDYSNTHSTRYVTVKDQWHSRGSTWLYRETCNLNCMVTITTARSKYPYHFFATSTGD
JHC            RNSQCYSSYSRVIAGTVFVAYHRDSYENKTMQLMLDDYSNTHSTRYVTVKDQWHSRGSTWLYRETCNLNCMVTITTARSKYPYHFFATSTGD
BE/13/2010     RNSQCYSSYSRVIAGTVFVAYHRDSYENKTMQLMLDDYSNTHSTRYVTVKDQWHSRGSTWLYRETCNLNCMVTITTARSKYPYHFFATSTGD
BE/35/2011     SHSQCYSSYSRVIAGTVFVAYHRDSYENKTMQLMLDDYSNTHSTRYVTVKDQWHSRGSTWLYRETCNLNCMVTITTARSKYPYHFFATSTGD
Toledo         SHSQCYSSYSRVIAGTVFVAYHRDSYENKTMQLMLDDYSNTHSIRYVTVKDQWHSRGSTWLYRETCNLNCMVTITTARSKYPYHFFATSTGD
C354A          RHTQCYSSYSRVIAGTVFVAYHRDSYENKTMQLMLDDYSNTHSTRYVTVKDQWHSRGSTWLYRETCNLNCMVTITTARSKYPYHFFATSTGD
C359A          RHSQCYSSYSRVIAGTVFVAYHRDSYENKTMQLMLDDYSNTHSTRYVTVKDQWHSRGSTWLYRETCNLNCMVTITTARSKYPYHFFATSTGD
BE/15/2011     RHSQCYSSYSRVIAGTVFVAYHRDSYENKTMQLMLDDYSNTHSTRYVTVKDQWHSRGSTWLYRETCNLNCMVTITTARSKYPYHFFATSTGD
HAN38          SHSQCYSSYSRVIAGTVFVAYHRDSYENKTMQLMLDDYSNTHSTRYVTVKDQWHSRGSTWLYRETCNLNCMVTITTARSKYPYHFFATSTGD
AD169          KFAQCYSSYSRVIGGTVFVAYHRDSYENKTMQLIPDDYSNTHSTRYVTVKDQWHSRGSTWLYRETCNLNCMLTITTARSKYPYHFFATSTGD
6397           KFAQCYSSYSRVIGGTVFVAYHRDSYENKTMQLIPDDYSNTHSTRYVTVKDQWHSRGSTWLYRETCNLNCMLTITTARSKYPYHFFATSTGD
BE/11/2010     KFAQCYSSYSRVIGGTVFVAYHRDSYENKTMQLIPDDYSNTHSTRYVTVKDQWHSRGSTWLYRETCNLNCMLTITTARSKYPYHFFATSTGD
C336A          KFAQCYSSYSRVIGGTVFVAYHRDSYENKTMQLMPDDYSNTHSTRYVTVKDQWHSRGSTWLYRETCNLNCMVTITTARSKYPYHFFATSTGD
HAN16          KFAQCYSSYSRVIGGTVFVAYHRDSYENKTMQLMSDDYSNTHSTRYVTVKDQWHSRGSTWLYRETCNLNCMVTITTARSKYPYHFFATSTGD
BE/16/2012     KFAQCYSSYSRVIGGTVFVAYHRDSYENKTMQLMPDDYSNTHSTRYVTVKDQWHSRGSTWLYRETCNLNCMVTITTARSKYPYHFFATSTGD
BE/15/2012     KFAQCYSSYSRVIGGTVFVAYHRDSYENKTMQLMPDDYSNTHSTRYVTVKDQWHSRGSTWLYRETCNLNCMVTITTARSKYPYHFFATSTGD
C338A          KFAQCYSSYSRVIGGTVFVAYHRDSYENKTMQLMPDDYSNTHSTRYVTVKDQWHSRGSTWLYRETCNLNCMVTITTARSKYPYHFFATSTGD
PAV21          KFAQCYSSYSRVIGGTVFVAYHRDSYENKTMQLMPDDYSNTHSTRYVTVKDQWHSRGSTWLYRETCNLNCMVTITTARSKYPYHFFATSTGD
C325A          SHSQCYSSYSRVIAGTVFVAYHRDSYENKTMQLMPDDYSNTHSTRYVTVKDQWHSRGSTWLYRETCNLNCMVTITTARSKYPYHFFATSTGD
BE/4/2012      SHSQCYSSYSRVIAGTVFVAYHRDSYENKTMQLMPDDYSNTHSTRYVTVKDQWHSRGSTWLYRETCNLNCMVTITTARSKYPYHFFATSTGD
HAN            SHSQCYSSYSRVIAGTVFVAYHRDSYENKTMQLMPDDYSNTHSTRYVTVKDQWHSRGSTWLYRETCNLNCMVTITTARSKYPYHFFATSTGD
7              SHSQCYSSYSRVIAGTVFVAYHRDSYENKTMQLMPDDYSNTHSTRYVTVKDQWHSRGSTWLYRETCNLNCMVTITTARSKYPYHFFATSTGD
BE/19/2011     SHSQCYSSYSRVIAGTVFVAYHRDSYENKTMQLMPDDYSNTHSTRYVTVKDQWHSRGSTWLYRETCNLNCMVTITTARSKYPYHFFATSTGD
C194A          SHSQCYSSYSRVIAGTVFVAYHRDSYENKTMQLMPDDYSNTHSTRYVTVKDQWHSRGSTWLYRETCNLNCMVTITTARSKYPYHFFATSTGD
BE/5/2012      SHSQCYSSYSRVIAGTVFVAYHRDSYENKTMQLMPDDYSNTHSTRYVTVKDQWHSRGSTWLYRETCNLNCMVTITTARSKYPYHFFATSTGD
CZ/2/2012      SHSQCYSSYSRVIAGTVFVAYHRDSYENKTMQLMLDDYSNTHSTRYVTVKDQWHSRGSTWLYRETCNLNCMVTITTARSKYPYHFFATSTGD
C128A          RHSQCYSSYSRVIAGTVFVAYHRDSYENKTMQLMPDDYSNTHSTRYVTVKDQWHSRGSTWLYRETCNLNCMVTITTARSKYPYHFFATSTGD
HAN2           SHSQCYSSYSRVIAGTVFVAYHRDSYENKTMQLMPDDYSNTHSTRYVTVKDQWHSRGSTWLYRETCNLNCMVTITTARSKYPYHFFATSTGD
CO82A          SHSQCYSSYSRVIAGTVFVAYHRDSYENKTMQLMPDDYSNTHSTRYVTVKDQWHSRGSTWLYRETCNLNCMVTITTARSKYPYHFFATSTGD
Merlin         SHSQCYSSYSRVIAGTVFVAYHRDSYENKTMQLMPDDYSNTHSTRYVTVKDQWHSRGSTWLYRETCNLNCMVTITTARSKYPYHFFATSTGD
BE/14/2011     SHSQCYSSYSRVIAGTVFVAYHRDSYENKTMQLMPDDYSNTHSTRYVTVKDQWHSRGSTWLYRETCNLNCMVTITTARSKYPYHFFATSTGD
BE/27/2010     SHSQCYSSYSRVIAGTVFVAYHRDSYENKTMQLMPDDYSNTHSTRYVTVKDQWHSRGSTWLYRETCNLNCMVTITTARSKYPYHFFATSTGD
Towne_varL     SHSQCYSSYSRVIAGTVFVAYHRDSYENKTMQLMPDDYSNTHSTRYVTVKDQWHSRGSTWLYRETCNLNCMVTITTARSKYPYHFFATSTGD
3157           SHSQCYSSYSRVIAGTVFVAYHRDSYENKTMQLMPDDYSNTHSTRYVTVKDQWHSRGSTWLYRETCNLNCMVTITTARSKYPYHFFATSTGD
Towne          SHSQCYSSYSRVIAGTVFVAYHRDSYENKTMQLMPDDYSNTHSTRYVTVKDQWHSRGSTWLYRETCNLNCMVTITTARSKYPYHFFATSTGD
BE/17/2010     SHSQCYSSYSRVIAGTVFVAYHRDSYENKTMQLMPDDYSNTHSTRYVTVKDQWHSRGSTWLYRETCNLNCMVTITTARSKYPYHFFATSTGD
BE/48/2011     SHSQCYSSYSRVIAGTVFVAYHRDSYENKTMQLMPDDYSNTHSTRYVTVKDQWHSRGSTWLYRETCNLNCMVTITTARSKYPYHFFATSTGD
TB40/E         SHSQCYSSYSRVIAGTVFVAYHRDSYENKTMQLMPDDYSNTHSTRYVTVKDQWHSRGSTWLYRETCNLNCMVTITTARSKYPYHFFATSTGN
BE/5/2011      RHSQCYSSYSRVIAGTVFVAYHRDSYENKTMQLMPDDYSNTHSTRYVTVKDQWHSRGSTWLYRETCNLNCMVTITTARSKYPYHFFATSTGD
BE/32/2011     GHSQCYSSYSRVIAGTVFVAYHRDSYENKTMQLMPDDYSNTHSTRYVTVKDQWHSRGSTWLYRETCNLNCMVTITTARSKYPYHFFATSTGD
C327A          SHSQCYSSYSRVIAGTVFVAYHRDSYENKTMQLMPDDYSNTHSTRYVTVKDQWHSRGSTWLYRETCNLNCMVTITTARSKYPYHFFATSTGD
C178A          SHSQCYSSYSRVIAGTVFVAYHRDSYENKTMQLMPDDYSNTHSTRYVTVKDQWHSRGSTWLYRETCNLNCMVTITTARSKYPYHFFATSTGD
BE/38/2011     SHSQCYSSYSRVIAGTVFVAYHRDSYENKTMQLMPDDYSNTHSTRYVTVKDQWHSRGSTWLYRETCNLNCMVTITTARSKYPYHFFATSTGD
HAN12          SHSQCYSSYSRVIAGTVFVAYHRDSYENKTMQLMPDDYSNTHSTRYVTVKDQWHSRGSTWLYRETCNLNCMVTITTARSKYPYHFFATSTGD
BE/13/2012     SHSQCYSSYSRVIAGTVFVAYHRDSYENKTMQLMPDDYSNTHSTRYVTVKDQWHSRGSTWLYRETCNLNCMVTITTARSKYPYHFFATSTGD
5510-E115      SHSQCYSSYSRVIAGTVFVAYHRDSYENKTMQLMPDDYSNTHSTRYVTVKDQWHSRGSTWLYRETCNLNCMVTITTARSKYPYHFFATSTGD
BE/22/2010     SHSQCYSSYSRVIAGTVFVAYHRDSYENKTMQLMPDDYSNTHSTRYVTVKDQWHSRGSTWLYRETCNLNCMVTITTARSKYPYHFFATSTGD
TR             SHSQCYSSYSRVIAGTVFVAYHRDSYENKTMQLMPDDYSNTHSTRYVTVKDQWHSRGSTWLYRETCNLNCMVTITTARSKYPYHFFATSTGD
BE/4/2010      KFAQCYSSYSRVIGGTVFVAYHRDSYENKTMQLIPDDYSNTHSTRYVTVKDQWHSRGSTWLYRETCNLNCMLTITTARSKYPYHFFATSTGD
JP             KFAQCYSSYSRVIGGTVFVAYHRDSYENKTMQLIPDDYSNTHSTRYVTVKDQWHSRGSTWLYRETCNLNCMLTITTARSKYPYHFFATSTGD
HAN19          KFAQCYSSYSRVIGGTVFVAYHRDSYENKTMQLIPDDYSNTHSTRYVTVKDQWHSRGSTWLYRETCNLNCMLTITTARSKYPYHFFATSTGD
UKNEQAS1       KFAQCYSSYSRVIAGTLFVAYHRDSYENKTMQLMSDDYSNTHSTRYVTVKDQWHSRGSTWLYHETCNLNCMVTMTAARSKYPYHFFATSTGD
S3             KFAQCYSSYSRVIAGTLFVAYHRDSYENKTMQLMSDDYSNTHSTRYVTVKDQWHSRGSTWLYHETCNLNCMVTMTAARSKYPYHFFATSTGD
consensus>70   .hsQCYSSYSRVIaGTvFVAYHRDSYENKTMQLm.DDYSNTHStRYVTVKDQWHSRGSTWLYrETCNLNCMvTiTtARSKYPYHFFATSTG#

                    280       290       300       310       320       330       340       350       360
HAN13          VVDISPFYNGTNRNTSYFGENADKFFIFPNYTIVSDFGRANSAPETHRLVAFLERADSVISWDIQDEKNVTCQLTFWEASERTIRSEAEDSY
BE/6/2011      VVDISPFYNGTNRNTSYFGENADKFFIFPNYTIVSDFGRANSAPETHRLVAFLERADSVISWDIQDEKNVTCQLTFWEASERTIRSEAEDSY
BE/37/2011     VVDISPFYNGTNRNTSYFGENADKFFIFPNYTIVSDFGRANSAPETHRLVAFLERADSVISWDIQDEKNVTCQLTFWEASERTIRSEAEDSY
VR1814         VVDISPFYNGTNRNTSYFGENADKFFIFPNYTIVSDFGRANSAPETHRLVAFLERADSVISWDIQDEKNVTCQLTFWEASERTIRSEAEDSY
HAN1           VVDISPFYNGTNRNTSYFGENADKFFIFPNYTIVSDFGRANSAPETHRLVAFLERADSVISWDIQDEKNVTCQLTFWEASERTIRSEAEDSY
CO76A          VVDISPFYNGTNRNTSYFGENADKFFIFPNYTIVSDFGRANSAPETHRLVAFLERADSVISWDIQDEKNVTCQLTFWEASERTIRSEAEDSY
HAN3           VVDISPFYNGTNRNTSYFGENADKFFIFPNYTIVSDFGRANSAPETHRLVAFLERADSVISWDIQDEKNVTCQLTFWEASERTIRSEAEDSY
BE/42/2011     VVDISPFYNGTNRNTSYFGENADKFFIFPNYTIVSDFGRANSAPETHRLVAFLERADSVISWDIQDEKNVTCQLTFWEASERTIRSEAEDSY
JHC            VVDISPFYNGTNRNTSYFGENADKFFIFPNYTIVSDFGRANSAPETHRLVAFLERADSVISWDIQDEKNVTCQLTFWEASERTIRSEAEDSY
BE/13/2010     VVDISPFYNGTNRNTSYFGENADKFFIFPNYTIVSDFGRANSAPETHRLVAFLERADSVISWDIQDEKNVTCQLTFWEASERTIRSEAEDSY
BE/35/2011     VVDISPFYNGTNRNTSYFGENADKFFIFPNYTIVSDFGRANSAPETHRLVAFLERADSVISWDIQDEKNVTCQLTFWEASERTIRSEAEDSY
Toledo         VVDISPFYNGTNRNTSYFGENADKFFIFPNYTIVSDFGRANSAPETHRLVAFLERADSVISWDIQDEKNVTCQLTFWEASERTIRSEAEDSY
C354A          VVDISPFYNGTNRNTSYFGENADKFFIFPNYTIVSDFGRANSAPETHRLVAFLERADSVISWDIQDEKNVTCQLTFWEASERTIRSEAEDSY
C359A          VVDISPFYNGTNRNTSYFGENADKFFIFPNYTIVSDFGRANSAPETHRLVAFLERADSVISWDIQDEKNVTCQLTFWEASERTIRSEAEDSY
BE/15/2011     VVDISPFYNGTNRNTSYFGENADKFFIFPNYTIVSDFGRANSAPETHRLVAFLERADSVISWDIQDEKNVTCQLTFWEASERTIRSEAEDSY
HAN38          VVDISPFYNGTNRNTSYFGENADKFFIFPNYTIVSDFGRANSAPETHRLVAFLERADSVISWDIQDEKNVTCQLTFWEASERTIRSEAEDSY
AD169          VVYISPFYNGTNRNASYFGENADKFFIFPNYTIVSDFGRPNAAPETHRLVAFLERADSVISWDIQDEKNVTCQLTFWEASERTIRSEAEDSY
6397           VVDISPFYNGTNRNASYFGENADKFFIFPNYTIVSDFGRPNAAPETHRLVAFLERADSVISWDIQDEKNVTCQLTFWEASERTIRSEAEDSY
BE/11/2010     VVDISPFYNGTNRNASYFGENADKFFIFPNYTIVSDFGRPNAAPETHRLVAFLERADSVISWDIQDEKNVTCQLTFWEASERTIRSEAEDSY
C336A          VVDISPFYNGTNRNASYFGENADKFFIFPNYTIVSDFGRPNAAPETHRLVAFLERADSVISWDIQDEKNVTCQLTFWEASERTIRSEAEDSY
HAN16          VVDISPFYNGTNRNASYFGENADKFFIFPNYTIVSDFGRPNAAPETHRLVAFLERADSVISWDIQDEKNVTCQLTFWEASERTIRSEAEDSY
BE/16/2012     VVDISPFYNGTNRNASYFGENADKFFIFPNYTIVSDFGRPNAAPETHRLVAFLERADSVISWDIQDEKNVTCQLTFWEASERTIRSEAEDSY
BE/15/2012     VVDISPFYNGTNRNASYFGENADKFFIFPNYTIVSDFGRPNAAPETHRLVAFLERADSVISWDIQDEKNVTCQLTFWEASERTIRSEAEDSY
C338A          VVDISPFYNGTNRNASYFGENADKFFIFPNYTIVSDFGRPNAAPETHRLVAFLERADSVISWDIQDEKNVTCQLTFWEASERTIRSEAEDSY
PAV21          VVDISPFYNGTNRNASYFGENADKFFIFPNYTIVSDFGRPNAAPETHRLVAFLERADSVISWDIQDEKNVTCQLTFWEASERTIRSEAEDSY
C325A          VVDISPFYNGTNRNASYFGENADKFFIFPNYTIVSDFGRPNSALETHRSVAFLERADSVISWDIQDEKNVTCQLTFWEASERTIRSEAEDSY
BE/4/2012      VVDISPFYNGTNRNASYFGENADKFFIFPNYTIVSDFGRPNSALETHRSVAFLERADSVISWDIQDEKNVTCQLTFWEASERTIRSEAEDSY
HAN            VVDISPFYNGTNRNASYFGENADKFFIFPNYTIVSDFGRPNSALETHRSVAFLERAESVISWDIQDEKNVTCQLTFWEASERTIRSEAEDSY
7              VVDISPFYNGTNRNASYFGENADKFFIFPNYTIVSDFGRPNSALETHRSVAFLERAESVISWDIQDEKNVTCQLTFWEASERTIRSEAEDSY
BE/19/2011     VVDISPFYNGTNRNASYFGENADKFFIFPNYTIVSDFGRPNSALETHRLVAFLERADSVISWDIQDEKNVTCQLTFWEASERTIRSEAEDSY
C194A          VVDISPFYNGTNRNASYFGENADKFFIFPNYTIVSDFGRPNSALETHRLVAFLERADSVISWDIQDEKNVTCQLTFWEASERTIRSEAEESY
BE/5/2012      VVDISPFYNGTNRNASYFGENADKFFIFPNYTIVSDFGRPNSALETHRLVAFLERADSVISWDIQDEKNVTCQLTFWEASERTIRSEAEESY
CZ/2/2012      VVDISPFYNGTNRNASYFGENADKFFIFPNYTIVSDFGRPNSALETHRLVAFLERADSVISWDIQDEKNVTCQLTFWEASERTIRSEAEESY
C128A          VVDISPFYNGTNRNASYFGENADKFFIFPNYTIVSDFGRPNSALETHRLVAFLERADSVISWDIQDEKNVTCQLTFWEASERTIRSEAEESY
HAN2           VVDISPFYNGTNRNASYFGENADKFFIFPNYTIVSDFGRPNSALETHRLVAFLERADSVISWDIQDEKNVTCQLTFWEASERTIRSEAEDSY
CO82A          VVDISPFYNGTNRNASYFGENADKFFIFPNYTIVSDFGRPNSALETHRLVAFLERADSVISWDIQDEKNVTCQLTFWEASERTIRSEAEDSY
Merlin         VVDISPFYNGTNRNASYFGENADKFFIFPNYTIVSDFGRPNSALETHRLVAFLERADSVISWDIQDEKNVTCQLTFWEASERTIRSEAEDSY
BE/14/2011     VVDISPFYNGTNRNASYFGENADKFFIFPNYTIVSDFGRPNSALETHRLVAFLERADSVISWDIQDEKNVTCQLTFWEASERTIRSEAEDSY
BE/27/2010     VVDISPFYNGTNRNASYFGENADKFFIFPNYTIVSDFGRPNSALETHRLVAFLERADSVISWDIQDEKNVTCQLTFWEASERTIRSEAEDSY
Towne_varL     VVDISPFYNGTNRNASYFGENADKFFIFPNYTIVSDFERPNSALETHRLVAFLERADSVISWDIQDEKNVTCQLTFWEASERTIRSEAEDSY
3157           VVDISPFYNGTNRNASYFGENADKFFIFPNYTIVSDFGRPNSALETHRLVAFLERADSVISWDIQDEKNVTCQLTFWEAPERTIRSEAEDSY
Towne          VVDISPFYNGTNRNASYFGENADKFFIFPNYTIVSDFGRPNSALETHRLVAFLERADSVISWDIQDEKNVTCQLTFWEASERTIRSEAEDSY
BE/17/2010     VVDISPFYNGTNRNASYFGENADKFFIFPNYTIVSDFGRPNSALETHRLVAFLERADSVISWDIQDEKNVTCQLTFWEASERTIRSEAEDSY
BE/48/2011     VVDISPFYNGTNRNASYFGENADKFFIFPNYTIVSDFGRPNSALETHRLVAFLERADSVISWDIQDEKNVTCQLTFWEASERTIRSEAEDSY
TB40/E         VVDISPFYNGTNRNASYFGENADKFFIFPNYTIVSDFGRPNSALETHRLVAFLERADSVISWDIQDEKNVTCQLTFWEASERTIRSEAEDSY
BE/5/2011      VVDISPFYNGTNRNASYFGENADKFFIFPNYTIVSDFGRPNSALETHRLVAFLERADSVISWDIQDEKNVTCQLTFWEASERTIRSEAEDSY
BE/32/2011     VVDISPFYNGTNRNASYFGENADKFFIFPNYTIVSDFGRPNSALETHRLVAFLERADSVISWDIQDEKNVTCQLTFWEASERTIRSEAEDSY
C327A          VVDISPFYNGTNRNASYFGENADKFFIFPNYTIVSDFGRPNSALETHRLVAFLERADSVISWDIQDEKNVTCQLTFWEASERTIRSEAEDSY
C178A          VVDISPFYNGTNRNASYFGENADKFFIFPNYTIVSDFGRPNSALETHRLVAFLERADSVISWDIQDEKNVTCQLTFWEASERTIRSEAEDSY
BE/38/2011     VVDISPFYNGTNRNASYFGENADKFFIFPNYTIVSDFGRPNSALETHRLVAFLERADSVISWDIQDEKNVTCQLTFWEASERTIRSEAEDSY
HAN12          VVDISPFYNGTNRNASYFGENADKFFIFPNYTIVSDFGRPNSALETHRLVAFLERADSVISWDIQDEKNVTCQLTFWEASERTIRSEAEDSY
BE/13/2012     VVDISPFYNGTNRNASYFGENADKFFIFPNYTIVSDFGRPNSALETHRLVAFLERADSVISWDIQDEKNVTCQLTFWEASERTIRSEAEDSY
5510-E115      VVDISPFYNGTNRNASYFGENADKFFIFPNYTIVSDFGRANSAPETHRLVAFLERADSVISWDIQDEKNVTCQLTFWEASERTIRSEAEDSY
BE/22/2010     VVDISPFYNGTNRNASYFGENADKFFIFPNYTIVSDFGRPNSALETHRLVAFLERADSVISWDIQDEKNVTCQLTFWEASERTIRSEAEDSY
TR             VVDISPFYNGTNRNASYFGENADKFFIFPNYTIVSDFGRPNSALETHRLVAFLERADSVISWDIQDEKNVTCQLTFWEASERTIRSEAEDSY
BE/4/2010      VVDISPFYNGTNRNASYFGENADKFFIFPNYTIVSDFGRPNAAPETHRLVAFLERADSVISWDIQDEKNVTCQLTFWEASERTIRSEAEDSY
JP             VVDISPFYNGTNRNASYFGENADKFFIFPNYTIVSDFGRPNAAPETHRLVAFLERADSVISWDIQDEKNVTCQLTFWEASERTIRSEAEDSY
HAN19          VVDISPFYNGTNRNASYFGENADKFFIFPNYTIVSDFGRPNAAPETHRLVAFLERADSVISWDIQDEKNVTCQLTFWEASERTIRSEAEDSY
UKNEQAS1       VVDISPFYNGTNRNTSYFGENADKFFIFPNYTIVSDFGRPNAAQETHRLVAFLERADSVISWDIQDEKNVTCQLTFWEASERTIRSEAKDSY
S3             VVDISPFYNGTNRNTSYFGENADKFFIFPNYTIVSDFGRPNAAQETHRLVAFLERADSVISWDIQDEKNVTCQLTFWEASERTIRSEAKDSY
consensus>70   VVdISPFYNGTNRNaSYFGENADKFFIFPNYTIVSDFgRpNsA.ETHRlVAFLERA#SVISWDIQDEKNVTCQLTFWEAsERTIRSEAe#SY
```

```
              370       380       390       400       410       420       430       440       450
HAN13         HFSSAKMTATFLSKKQEVNMSDPVLDCVRDQALNKLQQIFNASYNQTYEKYGNVSVFETTGGLVVFWQGIKQKSLLELERLANSSGVNSTR.
BE/6/2011     HFSSAKMTATFLSKKQEVNMSDPVLDCVRDQALNKLQQIFNASYNQTYEKYGNVSVFETTGGLVVFWQGIKQKSLLELERLANSSGVNSTR.
BE/37/2011    HFSSAKMTATFLSKKQEVNMSDPVLDCVRDQALNKLQQIFNASYNQTYEKYGNVSVFETTGGLVVFWQGIKQKSLLELERLANSSGVNSTR.
VR1814        HFSSAKMTATFLSKKQEVNMSDPVLDCVRDQALNKLQQIFNASYNQTYEKYGNVSVFETTGGLVVFWQGIKQKSLLELERLANSSGVNSTR.
HAN1          HFSSAKMTATFLSKKQEVNMSDPVLDCVRDQALNKLQQIFNASYNQTYEKYGNVSVFETTGGLVVFWQGIKQKSLLELERLANSSGVNSTR.
CO76A         HFSSAKMTATFLSKKQEVNMSDPVLDCVRDQALNKLQQIFNASYNQTYEKYGNVSVFETTGGLVVFWQGIKQKSLLELERLANSSGVNSTR.
HAN3          HFSSAKMTATFLSKKQEVNMSDPVLDCVRDQALNKLQQIFNASYNQTYEKYGNVSVFETTGGLVVFWQGIKQKSLLELERLANSSGVNSTR.
BE/42/2011    HFSSAKMTATFLSKKQEVNMSDPVLDCVRDQALNKLQQIFNASYNQTYEKYGNVSVFETTGGLVVFWQGIKQKSLLELERLANSSGVNSTR.
JHC           HFSSAKMTATFLSKKQEVNMSDPVLDCVRDQALNKLQQIFNASYNQTYEKYGNVSVFETTGGLVVFWQGIKQKSLLELERLANSSGVNSTR.
BE/13/2010    HFSSAKMTATFLSKKQEVNMSDPVLDCVRDQALNKLQQIFNASYNQTYEKYGNVSVFETTGGLVVFWQGIKQKSLLELERLANSSGVNSTR.
BE/35/2011    HFSSAKMTATFLSKKQEVNMSDPVLDCVRNQALNKLQQIFNASYNQTYEKYGNVSVFETTGGLVVFWQGIKQKSLLELERLANSSGVNSTR.
Toledo        HFSSAKMTATFLSKKQEVNMSDPVLDCVRNQALDKLQQIFNASYNQTYEKYGNVSVFETTGGLVVFWQGIKQKSLLELERLANSSGVNSTR.
C354A         HFSSAKMTATFLSKKQEVNMSDPVLDCVRNQALNKLQQIFNASYNQTYEKYGNVSVFETTGGLVVFWQGIKQKSLLELERLANSSGVNSTR.
C359A         HFSSAKMTATFLSKKQEVNMSDPMLDCVRDQALNKLQQIFNASYNQTYEKYGNVSVFETTGGLVVFWQGIKQKSLLELERLANSSGVNSTR.
BE/15/2011    HFSSAKMTATFLSKKQEVNMSDPVLDCVRDQALNKLQQIFNASYNQTYEKYGNVSVFETTGGLVVFWQGIKQKSLLELERLANSSGVNSTR.
HAN38         HFSSAKMTATFLSKKQEVNMSDPVLDCVRNQALNKLQQIFNASYNQTYEKYGNVSVFETTGGLVVFWQGIKQKSLLELERLANSSGVNSTR.
AD169         HFSSAKMTATFLSKKQEVNMSDSALDCVRDEAINKLQQIFNTSYNQTYEKYGNVSVFETSGGLVVFWQGIKQKSLVELERLANRSSLNITH.
6397          HFSSAKMTATFLSKKQEVNMSDSALDCVRDEAINKLQQIFNTSYNQTYEKYGNVSVFETSGGLVVFWQGIKQKSLVELERLANRSSLNITH.
BE/11/2010    HFSSAKMTATFLSKKQEVNMSDSALDCVRDEAINKLQQIFNTSYNQTYEKYGNVSVFETSGGLVVFWQGIKQKSLVELERLANRSSLNITH.
C336A         HFSSAKMTATFLSKKQEVNMSDSALDCVRDEAINKLQQIFNTSYNQTYEKYGNVSVFETSGGLVVFWQGIKQKSLVELERLANRSSLNITH.
HAN16         HFSSAKMTATFLSKKQEVNMSDSALDCVRDEAINKLQQIFNTSYNQTYEKYGNVSVFETSGGLVVFWQGIKQKSLVELERLANRSSLNITH.
BE/16/2012    HFSSAKMTATFLSKKQEVNMSDSALDCVRVEAINKLQQIFNTSYNQTYEKYGNVSVFETSGGLVVFWQGIKQKSLVELERLANRSSLNITH.
BE/15/2012    HFSSAKMTATFLSKKQEVNMSDSALDCVRVEAINKLQQIFNTSYNQTYEKYGNVSVFETSGGLVVFWQGIKQKSLVELERLANRSSLNITH.
C338A         HFSSAKMTATFLSKKQEVNMSDSALDCVRDEAINKLQQIFNTSYNQTYEKYGNVSVFETSGGLVVFWQGIKQKSLVELERLANRSSLNITH.
PAV21         HFSSAKMTATFLSKKQEVNMSDSALDCVRDEAINKLQQIFNTSYNQTYEKYGNVSVFETSGGLVVFWQGIKQKSLVELERLANRSSLNITH.
C325A         HFSSAKMTATFLSKKQEVNMSDSALDCVRDEAINKLQQIFNTSYNQTYEKYGNVSVFETTGGLVVFWQGIKQKSLVELERLANRSSLNLTHN
BE/4/2012     HFSSAKMTATFLSKKQEVNMSDSALDCVRDEAINKLQQIFNTSYNQTYEKYGNVSVFETTGGLVVFWQGIKQKSLVELERLANRSSLNLTHN
HAN           HFSSAKMTATFLSKKQEVNMSDSALDCVRDEAINKLQQIFNTSYNQTYEKYGNVSVFETTGGLVVFWQGIKQKSLVELERLANRSSLNLTHN
7             HFSSAKMTATFLSKKQEVNMSDSALDCVRDEAINKLQQIFNTSYNQTYEKYGNVSVFETTGGLVVFWQGIKQKSLVELERLANRSSLNLTHN
BE/19/2011    HFSSAKMTATFLSKKQEVNMSDSALDCVRDEAINKLQQIFNASYNQTYEKYGNVSVFETTGGLVVFWQGIKQKSLVELERLANRSSLNLTHN
C194A         HFSSAKMTATFLSKKQEVNMSDSALDCVRDEALNKLQQIFNASYNQTYEKYGNVSVFETTGGLVVFWQGIKQKSLVELERLANRSSLNLTHS
BE/5/2012     HFSSAKMTATFLSKKQEVNMSDSALDCVRDEALNKLQQIFNASYNQTYEKYGNVSVFETTGGLVVFWQGIKQKSLVELERLANRSSLNLTHS
CZ/2/2012     HFSSAKMTATFLSKKQEVNMSDSALDCVRDEALNKLQQIFNASYNQTYEKYGNVSVFETTGGLVVFWQGIKQKSLVELERLANRSSLNLTHS
C128A         HFSSAKMTATFLSKKQEVNMSDSALDCVRDEALNKLQQIFNTSYNQTYEKYGNVSVFETTGGLVVFWQGIKQKSLVELERLANRSSLNLTHS
HAN2          HFSSAKMTATFLSKKQEVNMSDSALDCVRDEAINKLQQIFNTSYNQTYEKYGNVSVFETTGGLVVFWQGIKQKSLVELERLANRSSLNLTHN
CO82A         HFSSAKMTATFLSKKQEVNMSDSALDCVRDEAINKLQQIFNTSYNQTYEKYGNVSVFETTGGLVVFWQGIKQKSLVELERLANRSSLNLTHN
Merlin        HFSSAKMTATFLSKKQEVNMSDSALDCVRDEAINKLQQIFNTSYNQTYEKYGNVSVFETTGGLVVFWQGIKQKSLVELERLANRSSLNLTHN
BE/14/2011    HFSSAKMTATFLSKKQEVNMSDSALDCVRDEAINKLQQIFNTSYNQTYEKYGNVSVFETTGGLVVFWQGIKQKSLVELERLANRSSLNLTHN
BE/27/2010    HFSSAKMTATFLSKKQEVNMSDSALDCVRDEAINKLQQIFNTSYNQTYEKYGNVSVFETTGGLVVFWQGIKQKSLVELERLANRSSLNLTHN
Towne_varL    HFSSAKMTATFLSKKQEVNMSDSALDCVRDEAINKLQQIFNTSYNQTYEKYGNVSVFETTGGLVVFWQGIKQKSLVELERLANRSSLNLTHN
3157          HFSSAKMTATFLSKKQEVNMSDSALDCVRDEAINKLQQIFNTSYNQTYEKYGNVSVFETTGGLVVFWQGIKQKSLVELERLANRSSLNLTHN
Towne         HFSSAKMTATFLSKKQEVNMSDSALDCVRDEAINKLQQIFNTSYNQTYEKYGNVSVFETTGGLVVFWQGIKQKSLVELERLANRSSLNLTHN
BE/17/2010    HFSSAKMTATFLSKKQEVNMSDSALDCVRDEAINKLQQIFNTSYNQTYEKYGNVSVFETTGGLVVFWQGIKQKSLVELERLANRSSLNLTHN
BE/48/2011    HFSSAKMTATFLSKKQEVNMSDSALDCVRDEAINKLQQIFNTSYNQTYEKYGNVSVFETTGGLVVFWQGIKQKSLVELERLANRSSLNLTHN
TB40/E        HFSSAKMTATFLSKKQEVNMSDSALDCVRDEAINKLQQIFNTSYNQTYEKYGNVSVFETTGGLVVFWQGIKQKSLVELERLANRSSLNLTHN
BE/5/2011     HFSSAKMTATFLSKKQEVNMSDSALDCVRDEAINKLQQIFNTSYNQTYEKYGNVSVFETTGGLVVFWQGIKQKSLVELERLANRSSLNLTHN
BE/32/2011    HFSSAKMTATFLSKKQEVNMSDSALDCVRDEAINKLQQIFNTSYNQTYEKYGNVSVFETTGGLVVFWQGIKQKSLVELERLANRSSLNLTHN
C327A         HFSSAKMTATFLSKKQEVNMSDSVLDCVRDEAINKLQQIFNTSYNQTYEKYGNVSVFETTGGLVVFWQGIKQKSLVELERLANRSSLNLTHN
C178A         HFSSAKMTATFLSKKQEVNMSDSVLDCVRDEAINKLQQIFNTSYNQTYEKYGNVSVFETTGGLVVFWQGIKQKSLVELERLANRSSLNLTHN
BE/38/2011    HFSSAKMTATFLSKKQEVNMSDSALDCVRDEAINKLQQIFNTSYNQTYEKYGNVSVFETTGGLVVFWQGIKQKSLVELERLANRSSLNLTHN
HAN12         HFSSAKMTATFLSKKQEVNMSDSALDCVRDEAINKLQQIFNTSYNQTYEKYGNVSVFETTGGLVVFWQGIKQKSLVELERLANRSSLNLTHN
BE/13/2012    HFSSAKMTATFLSKKQEVNMSDSALDCVRDEAINKLQQIFNTSYNQTYEKYGNVSVFETTGGLVVFWQGIKQKSLVELERLANRSSLNLTHN
5510-E115     HFSSAKMTATFLSKKQEVNMSDSVLDCVRDEAINKLQQIFNTSYNQTYEKYGNVSVFETTGGLVVFWQGIKQKSLVELERLANRSSLNLTHS
BE/22/2010    HFSSAKMTATFLSKKQEVNMSDSALDCVRDEAINKLQQIFNTSYNQTYEKYGNVSVFETTGGLVVFWQGIKQKSLVELERLANRSSLNLTHS
TR            HFSSAKMTATFLSKKQEVNMSDSALDCVRDEAINKLQQIFNTSYNQTYEKYGNVSVFETTGGLVVFWQGIKQKSLVELERLANRSSLNLTHS
BE/4/2010     HFSSAKMTATFLSKKQEVNMSDSALDCVRDEAINKLQQIFNTSYNQTYEKYGNVSVFETSGGLVVFWQGIKQKSLVELERLANRSSLNITH.
JP            HFSSAKMTATFLSKKQEVNMSDSALDCVRDEAINKLQQIFNTSYNQTYEKYGNVSVFETSGGLVVFWQGIKQKSLVELERLANRSSLNITH.
HAN19         HFSSAKMTATFLSKKQEVNMSDSALDCVRDEAINKLQQIFNTSYNQTYEKYGNVSVFETSGGLVVFWQGIKQKSLVELERLANRSSLNITH.
UKNEQAS1      HFSSAKMTATFLSKKQEVNMSDPVLDCVRDQALNKLQQIFNASYNQTYEKYGNVSVFETTGGLVVFWQGIKQKSLLELERLANSSGVNATR.
S3            HFSSAKMTATFLSKKQEVNMSDPVLDCVRDQALNKLQQIFNASYNQTYEKYGNVSVFETTGGLVVFWQGIKQKSLLELERLANSSGVNATR.
consensus>70  HFSSAKMTATFLSKKQEVNMSDs.LDCVRd#A.#KLQQIFN.SYNQTYEKYGNVSVFETtGGLVVFWQGIKQKSLvELERLANrSslN.Th.

                 460       470       480       490       500       510       520       530       540
HAN13         RTKRST.GNTTTLSLESESVRNVLYAQLQFTYDTLRSYINRALAQIAEAWCVDQRRTLEVFKELSKINPSAILSAIYNKPIAARFMGDVLGL
BE/6/2011     RTKRST.GNTTTLSLESESVRNVLYAQLQFTYDTLRSYINRALAQIAEAWCVDQRRTLEVFKELSKINPSAILSAIYNKPIAARFMGDVLGL
BE/37/2011    RTKRST.GNTTTLSLESESVRNVLYAQLQFTYDTLRSYINRALAQIAEAWCVDQRRTLEVFKELSKINPSAILSAIYNKPIAARFMGDVLGL
VR1814        RTKRST.GNTTTLSLESESVRNVLYAQLQFTYDTLRSYINRALAQIAEAWCVDQRRTLEVFKELSKINPSAILSAIYNKPIAARFMGDVLGL
HAN1          RTKRST.GNTTTLSLESESVRNVLYAQLQFTYDTLRSYINRALAQIAEAWCVDQRRTLEVFKELSKINPSAILSAIYNKPIAARFMGDVLGL
CO76A         RTKRST.GNTTTLSLESESVRNVLYAQLQFTYDTLRSYINRALAQIAEAWCVDQRRTLEVFKELSKINPSAILSAIYNKPIAARFMGDVLGL
HAN3          RTKRST.GNTTTLSLESESVRNVLYAQLQFTYDTLRSYINRALAQIAEAWCVDQRRTLEVFKELSKINPSAILSAIYNKPIAARFMGDVLGL
BE/42/2011    RTKRST.GNTTTLSLESESVRNVLYAQLQFTYDTLRSYINRALAQIAEAWCVDQRRTLEVFKELSKINPSAILSAIYNKPIAARFMGDVLGL
JHC           RTKRST.GNTTTLSLESESVRNVLYAQLQFTYDTLRSYINRALAQIAEAWCVDQRRTLEVFRELSKINPSAILSAIYNKPIAARFMGDVLGL
BE/13/2010    RTKRST.GNTTTLSLESESVRNVLYAQLQFTYDTLRSYINRALAQIAEAWCVDQRRTLEVFRELSKINPSAILSAIYNKPIAARFMGDVLGL
BE/35/2011    RTKRST.GNTTTLSLESESVRNVLYAQLQFTYDTLRSYINRALAQIAEAWCVDQRRTLEVFKELSKINPSAILSAIYNKPIAARFMGDVLGL
Toledo        RTKRST.GNTTTLSPESESVRNVLYAQLQFTYDTLRSYINRALAQIAEAWCVDQRRTLEVFKELSKINPSAILSAIYNKPIAARFMGDVLGL
C354A         RTKRST.GNTTTLSLESESVRNVLYAQLQFTYDTLRSYINRALAQIAEAWCVDQRRTLEVFKELSKINPSAILSAIYNKPIAARFMGDVLGL
C359A         RTKRST.GNTTTLSPESESVRNVLYAQLQFTYDTLRSYINRALAQIAEAWCVDQRRTLEVFKELSKINPSAILSAIYNKPIAARFMGDVLGL
BE/15/2011    RTKRST.GNTTTLSLENESVRNVLYAQLQFTYDTLRSYINRALAQIAEAWCVDQRRTLEVFKELSKINPSAILSAIYNKPIAARFMGDVLGL
HAN38         RTKRST.GNTTTLSLESESVRNVLYAQLQFTYDTLRSYINRALAQIAEAWCVDQRRTLEVFKELSKINPSAILSAIYNKPIAARFMGDVLGL
AD169         RTRRSTSDNNTTHLSSMESVHNLVYAQLQFTYDTLRGYINRALAQIAEAWCVDQRRTLEVFKELSKINPSAILSAIYNKPIAARFMGDVLGL
6397          RTRRSTSDNNTTHLSSMESVHNLVYAQLQFTYDTLRGYINRALAQIAEAWCVDQRRTLEVFKELSKINPSAILSAIYNKPIAARFMGDVLGL
BE/11/2010    RTRRSTSDNNTTHLSSMESVHNLVYAQLQFTYDTLRGYINRALAQIAEAWCVDQRRTLEVFKELSKINPSAILSAIYNKPIAARFMGDVLGL
C336A         RTRRSTSDNNTTHLSSMESVHNLVYAQLQFTYDTLRGYINRALAQIAEAWCVDQRRTLEVFKELSKINPSAILSAIYNKPIAARFMGDVLGL
HAN16         RTRRSTSDNNTTHLSSMESVHNLVYAQLQFTYDTLRGYINRALAQIAEAWCVDQRRTLEVFKELSKINPSAILSAIYNKPIAARFMGDVLGL
BE/16/2012    RTRRSTSDNNTTHLSSMESVHNLVYAQLQFTYDTLRGYINRALAQIAEAWCVDQRRTLEVFKELSKINPSAILSAIYNKPIAARFMGDVLGL
BE/15/2012    RTRRSTSDNNTTHLSSMESVHNLVYAQLQFTYDTLRGYINRALAQIAEAWCVDQRRTLEVFKELSKINPSAILSAIYNKPIAARFMGDVLGL
C338A         RTRRSTSDNNTTHLSSMESVHNLVYAQLQFTYDTLRGYINRALAQIAEAWCVDQRRTLEVFKELSKINPSAILSAIYNKPIAARFMGDVLGL
PAV21         RTRRSTSDNNTTHLSSMESVHNLVYAQLQFTYDTLRGYINRALAQIAEAWCVDQRRTLEVFKELSKINPSAILSAIYNKPIAARFMGDVLGL
C325A         RTKRSTNGNNATHLSNMESVHNLVYAQLQFTYDTLRGYINRALAQIAEAWCVDQRRSLEVFRELSKINPSAILSAIYNKPIAARFMGDVLGL
BE/4/2012     RTKRSTNGNNATHLSNMESVHNLVYAQLQFTYDTLRGYINRALAQIAEAWCVDQRRSLEVFRELSKINPSAILSAIYNKPIAARFMGDVLGL
HAN           RTKRSTNGSNATHLSNMESVHNLVYAQLQFTYDTLRGYINRALAQIAEAWCVDQRRSLEVFRELSKINPSAILSAIYNKPIAARFMGDVLGL
7             RTKRSTNGSNATHLSNMESVHNLVYAQLQFTYDTLRGYINRALAQIAEAWCVDQRRSLEVFRELSKINPSAILSAIYNKPIAARFMGDVLGL
BE/19/2011    RTKRSTDGNNATHLSNMESVHNLVYAQLQFTYDTLRGYINRALAQIAEAWCVDQRRTLEVFKELSKINPSAILSAIYNKPIAARFMGDVLGL
C194A         RTRRSTDGTNVTHLSNMDSVHNLVYAQLQFTYDTLRGYINRALTQIAEAWCVDQRRTLEVFKELSKINPSAILSAIYNKPIAARFMGDVLGL
BE/5/2012     RTRRSTDGTNVTHLSNMDSVHNLVYAQLQFTYDTLRGYINRALTQIAEAWCVDQRRTLEVFKELSKINPSAILSAIYNKPIAARFMGDVLGL
CZ/2/2012     RTRRSTDGTNVTHLSNMDSVHNLVYAQLQFTYDTLRGYINRALTQIAEAWCVDQRRTLEVFKELSKINPSAILSAIYNKPIAARFMGDVLGL
C128A         RTRRSTDGTNVTHLSNMDSVHNLVYAQLQFTYDTLRGYINRALTQIAEAWCVDQRRTLEVFKELSKINPSAILSAIYNKPIAARFMGDVLGL
HAN2          RTKRSTDGNNATHLSNMESVHNLVYAQLQFTYDTLRGYINRALAQIAEAWCVDQRRTLEVFKELSKINPSAILSAIYNKPIAARFMGDVLGL
CO82A         RTKRSTDGNNATHLSNMESVHNLVYAQLQFTYDTLRGYINRALAQIAEAWCVDQRRTLEVFKELSKINPSAILSAIYNKPIAARFMGDVLGL
Merlin        RTKRSTDGNNATHLSNMESVHNLVYAQLQFTYDTLRGYINRALAQIAEAWCVDQRRTLEVFKELSKINPSAILSAIYNKPIAARFMGDVLGL
BE/14/2011    RTKRSTDGNNATHLSNMESVHNLVYAQLQFTYDTLRGYINRALAQIAEAWCVDQRRTLEVFKELSKINPSAILSAIYNKPIAARFMGDVLGL
BE/27/2010    RTKRSTDGNNATHLSNMESVHNLVYAQLQFTYDTLRGYINRALAQIAEAWCVDQRRTLEVFKELSKINPSAILSAIYNKPIAARFMGDVLGL
Towne_varL    RTKRSTDGNNATHLSNMESVHNLVYAQLQFTYDTLRGYINRALAQIAEAWCVDQRRTLEVFKELSKINPSAILSAIYNKPIAARFMGDVLGL
3157          RTKRSTDGNNATHLSNMESVHNLVYAQLQFTYDTLRGYINRALAQIAEAWCVDQRRTLEVFKELSKINPSAILSAIYNKPIAARFMGDVLGL
Towne         RTKRSTDGNNATHLSNMESVHNLVYAQLQFTYDTLRGYINRALAQIAEAWCVDQRRTLEVFKELSKINPSAILSAIYNKPIAARFMGDVLGL
BE/17/2010    RTKRSTDGNNATHLSNMESVHNLVYAQLQFTYDTLRGYINRALAQIAEAWCVDQRRTLEVFKELSKINPSAILSAIYNKPIAARFMGDVLGL
BE/48/2011    RTKRSTDGNNATHLSNMESVHNLVYAQLQFTYDTLRGYINRALAQIAEAWCVDQRRTLEVFKELSKINPSAILSAIYNKPIAARFMGDVLGL
TB40/E        RTKRSTDGNNATHLSNMESVHNLVYAQLQFTYDTLRGYINRALAQIAEAWCVDQRRTLEVFKELSKINPSAILSAIYNKPIAARFMGDVLGL
BE/5/2011     RTKRSTDGNNATHLSNMESVHNLVYAQLQFTYDTLRGYINRALAQIAEAWCVDQRRTLEVFKELSKINPSAILSAIYNKPIAARFMGDVLGL
BE/32/2011    RTKRSTDGNNATHLSNMESVHNLVYAQLQFTYDTLRGYINRALAQIAEAWCVDQRRTLEVFKELSKINPSAILSAIYNKPIAARFMGDVLGL
C327A         RTKRSTDGNNATHLSNMESVHNLVYAQLQFTYDTLRGYINRALAQIAEAWCVDQRRTLEVFKELSKINPSAILSAIYNKPIAARFMGDVLGL
C178A         RTKRSTDGNNATHLSNMESVHNLVYAQLQFTYDTLRGYINRALAQIAEAWCVDQRRTLEVFKELSKINPSAILSAIYNKPIAARFMGDVLGL
BE/38/2011    RTKRSIDGNNATHLSNMESVHNLVYAQLQFTYDTLRGYINRALAQIAEAWCVDQRRTLEVFKELSKINPSAILSAIYNKPIAARFMGDVLGL
HAN12         RTKRSIDGNNATHLSNMESVHNLVYAQLQFTYDTLRGYINRALAQIAEAWCVDQRRTLEVFKELSKINPSAILSAIYNKPIAARFMGDVLGL
BE/13/2012    RTKRSTDGNNATHLSNMESVHNLVYAQLQFTYDTLRGYINRALAQIAEAWCVDQRRSLEVFKELSKINPSAILSAIYNKPIAARFMGDVLGL
5510-E115     RAKRSADGNNTTHLSNMESVHNLVYAQLQFTYDTLRGYINRALAQIAEAWCVDQRRSLEVFRELSKINPSAILSAIYNKPIAARFMGDVLGL
BE/22/2010    RTKRSTDGNNATHLSNMESVHNLVYAQLQFTYDTLRGYINRALAQIAEAWCVDQRRSLEVFRELSKINPSAILSAIYNKPIAARFMGDVLGL
TR            RTKRSADGNNATHLSSMESVHNLVYAQLQFTYDTLRGYINRALAQIAEAWCVDQRRSLEVFKELSKINPSAILSAIYNKPIAARFMGDVLGL
BE/4/2010     RTRRSTSDNNTTHLSSMESVHNLVYAQLQFTYDTLRGYINRALAQIAEAWCVDQRRTLEVFKELSKINPSAILSAIYNKPIAARFMGDVLGL
JP            RTRRSTSDNNTTHLSSMESVHNLVYAQLQFTYDTLRGYINRALAQIAEAWCVDQRRTLEVFKKLSKINPSAILSAIYNKPIAARFMGDVLGL
HAN19         RTRRSTSDNNTTHLSSMESVHNLVYAQLQFTYDTLRGYINRALAQIAEAWCVDQRRTLEVFKELSKINPSAILSAIYNKPIAARFMGDVLGL
UKNEQAS1      RSKRSTNNTTTLSLEN.DSVRSVLYAQLQFTYDTLRNYINRALAQIAEAWCVDQRRTLEVFKELSKINPSAILSAIYNKPIAARFMGDVLGL
S3            RSKRSTNNTTTLSLEN.DSVRSVLYAQLQFTYDTLRNYINRALAQIAEAWCVDQRRTLEVFKELSKINPSAILSAIYNKPIAARFMGDVLGL
consensus>70  RtkRSt.gnn.thlsnm#SVhnlvYAQLQFTYDTLRgYINRALaQIAEAWCVDQRRtLEVFkeLSKINPSAILSAIYNKPIAARFMGDVLGL
```

```
              550       560       570       580       590       600       610       620       630
HAN13         ASCVTINQTSVKVLRDMNVKESPGRCYSRPVVIFNFVNSSYVQYGQLGEDNEILLGNHRTEECQFPSLKIFIAGNSAYEYVDYLFKRMIDLS
BE/6/2011     ASCVTINQTSVKVLRDMNVKESPGRCYSRPVVIFNFVNSSYVQYGQLGEDNEILLGNHRTEECQFPSLKIFIAGNSAYEYVDYLFKRMIDLS
BE/37/2011    ASCVTINQTSVKVLRDMNVKESPGRCYSRPVVIFNFVNSSYVQYGQLGEDNEILLGNHRTEECQFPSLKIFIAGNSAYEYVDYLFKRMIDLS
VR1814        ASCVTINQTSVKVLRDMNVKESPGRCYSRPVVIFNFVNSSYVQYGQLGEDNEILLGNHRTEECQFPSLKIFIAGNSAYEYVDYLFKRMIDLS
HAN1          ASCVTINQTSVKVLRDMNVKESPGRCYSRPVVIFNFVNSSYVQYGQLGEDNEILLGNHRTEECQFPSLKIFIAGNSAYEYVDYLFKRMIDLS
CO76A         ASCVTINQTSVKVLRDMNVKESPGRCYSRPVVIFNFVNSSYVQYGQLGEDNEILLGNHRTEECQFPSLKIFIAGNSAYEYVDYLFKRMIDLS
HAN3          ASCVTINQTSVKVLRDMNVKESPGRCYSRPVVIFNFVNSSYVQYGQLGEDNEILLGNHRTEECQFPSLKIFIAGNSAYEYVDYLFKRMIDLS
BE/42/2011    ASCVTINQTSVKVLRDMNVKESPGRCYSRPVVIFNFVNSSYVQYGQLGEDNEILLGNHRTEECQFPSLKIFIAGNSAYEYVDYLFKRMIDLS
JHC           ASCVTINQTSVKVLRDMNVKESPGRCYSRPVVIFNFVNSSYVQYGQLGEDNEILLGNHRTEECQFPSLKIFIAGNSAYEYVDYLFKRMIDLS
BE/13/2010    ASCVSINQTSVKVLRDMNVKESPGRCYSRPVVIFNFVNSSYVQYGQLGEDNEILLGNHRTEECQFPSLKIFIAGNSAYEYVDYLFKRMIDLS
BE/35/2011    ASCVTINQTSVKVLRDMNVKESPGRCYSRPVVIFNFVNSSYVQYGQLGEDNEILLGNHRTEECQFPSLKIFIAGNSAYEYVDYLFKRMIDLS
Toledo        ASCVTINQTSVKVLRDMNVKESPGRCYSRPVVIFNFVNSSYVQYGQLGEDNEILLGNHRTEECQFPSLKIFIAGNSAYEYVDYLFKRMIDLS
C354A         ASCVTINQTSVKVLRDMNVKESPGRCYSRPVVIFNFVNSSYVQYGQLGEDNEILLGNHRTEECQFPSLKIFIAGNSAYEYVDYLFKRMIDLS
C359A         ASCVTINQTSVKVLRDMNVKESPGRCYSRPVVIFNFVNSSYVQYGQLGEDNEILLGNHRTEECQFPSLKIFIAGNSAYEYVDYLFKRMIDLS
BE/15/2011    ASCVTINQTSVKVLRDMNVKESPGRCYSRPVVIFNFVNSSYVQYGQLGEDNEILLGNHRTEECQFPSLKIFIAGNSAYEYVDYLFKRMIDLS
HAN38         ASCVTINQTSVKVLRDMNVKESPGRCYSRPVVIFNFVNSSYVQYGQLGEDNEILLGNHRTEECQFPSLKIFIAGNSAYEYVDYLFKRMIDLS
AD169         ASCVTINQTSVKVLRDMNVKESPGRCYSRPVVIFNFANSSYVQYGQLGEDNEILLGNHRTEECQLPSLKIFIAGNSAYEYVDYLFKRMIDLS
6397          ASCVTINQTSVKVLRDMNVKESPGRCYSRPVVIFNFANSSYVQYGQLGEDNEILLGNHRTEECQLPSLKIFIAGNSAYEYVDYLFKRMIDLS
BE/11/2010    ASCVTINQTSVKVLRDMNVKESPGRCYSRPVVIFNFANSSYVQYGQLGEDNEILLGNHRTEECQLPSLKIFIAGNSAYEYVDYLFKRMIDLS
C336A         ASCVTINQTSVKVLRDMNVKESPGRCYSRPVVIFNFANSSYVQYGQLGEDNEILLGNHRTEECQLPSLKIFIAGNSAYEYVDYLFKRMIDLS
HAN16         ASCVTINQTSVKVLRDMNVKESPGRCYSRPVVIFNFANSSYVQYGQLGEDNEILLGNHRTEECQLPSLKIFIAGNSAYEYVDYLFKRMIDLS
BE/16/2012    ASCVTINQTSVKVLRDMNVKESPGRCYSRPVVIFNFANSSYVQYGQLGEDNEILLGNHRTEECQLPSLKIFIAGNSAYEYVDYLFKRMIDLS
BE/15/2012    ASCVTINQTSVKVLRDMNVKESPGRCYSRPVVIFNFANSSYVQYGQLGEDNEILLGNHRTEECQLPSLKIFIAGNSAYEYVDYLFKRMIDLS
C338A         ASCVTINQTSVKVLRDMNVKESPGRCYSRPVVIFNFANSSYVQYGQLGEDNEILLGNHRTEECQLPSLKIFIAGNSAYEYVDYLFKRMIDLS
PAV21         ASCVTINQTSVKVLRDMNVKESPGRCYSRPVVIFNFANSSYVQYGQLGEDNEILLGNHRTEECQLPSLKIFIAGNSAYEYVDYLFKRMIDLS
C325A         ASCVTINQTSVKVLRDMNVKESPGRCYSRPVVIFNFANSSYVQYGQLGEDNEILLGNHRTEECQLPSLKIFIAGNSAYEYVDYLFKRMIDLS
BE/4/2012     ASCVTINQTSVKVLRDMNVKESPGRCYSRPVVIFNFANSSYVQYGQLGEDNEILLGNHRTEECQLPSLKIFIAGNSAYEYVDYLFKRMIDLS
HAN           ASCVTINQTSVKVLRDMNVKESPGRCYSRPVVIFNFANSSYVQYGQLGEDNEILLGNHRTEECQLPSLKIFIAGNSAYEYVDYLFKRMIDLS
7             ASCVTINQTSVKVLRDMNVKESPGRCYSRPVVIFNFANSSYVQYGQLGEDNEILLGNHRTEECQLPSLKIFIAGNSAYEYVDYLFKRMIDLS
BE/19/2011    ASCVTINQTSVKVLRDMNVKESPGRCYSRPVVIFNFANSSYVQYGQLGEDNEILLGNHRTEECQLPSLKIFIAGNSAYEYVDYLFKRMIDLS
C194A         ASCVTINQTSVKVLRDMNVKESPGRCYSRPVVIFNFANSSYVQYGQLGEDNEILLGNHRTEECQLPSLKIFIAGNSAYEYVDYLFKRMIDLS
BE/5/2012     ASCVTINQTSVKVLRDMNVKESPGRCYSRPVVIFNFANSSYVQYGQLGEDNEILLGNHRTEECQLPSLKIFIAGNSAYEYVDYLFKRMIDLS
CZ/2/2012     ASCVTINQTSVKVLRDMNVKESPGRCYSRPVVIFNFANSSYVQYGQLGEDNEILLGNHRTEECQLPSLKIFIAGNSAYEYVDYLFKRMIDLS
C128A         ASCVTINQTSVKVLRDMNVKESPGRCYSRPVVIFNFANSSYVQYGQLGEDNEILLGNHRTEECQLPSLKIFIAGNSAYEYVDYLFKRMIDLS
HAN2          ASCVTINQTSVKVLRDMNVKESPGRCYSRPVVIFNFANSSYVQYGQLGEDNEILLGNHRTEECQLPSLKIFIAGNSAYEYVDYLFKRMIDLS
CO82A         ASCVTINQTSVKVLRDMNVKESPGRCYSRPVVIFNFANSSYVQYGQLGEDNEILLGNHRTEECQLPSLKIFIAGNSAYEYVDYLFKRMIDLS
Merlin        ASCVTINQTSVKVLRDMNVKESPGRCYSRPVVIFNFANSSYVQYGQLGEDNEILLGNHRTEECQLPSLKIFIAGNSAYEYVDYLFKRMIDLS
BE/14/2011    ASCVTINQTSVKVLRDMNVKESPGRCYSRPVVIFNFANSSYVQYGQLGEDNEILLGNHRTEECQLPSLKIFIAGNSAYEYVDYLFKRMIDLS
BE/27/2010    ASCVTINQTSVKVLRDMNVKESPGRCYSRPVVIFNFANSSYVQYGQLGEDNEILLGNHRTEECQLPSLKIFIAGNSAYEYVDYLFKRMIDLS
Towne_varL    ASCVTINQTSVKVLRDMNVKESPGRCYSRPVVIFNFANSSYVQYGQLGEDNEILLGNHRTEECQLPSLKIFIAGNSAYEYVDYLFKRMIDLS
3157          ASCVTINQTSVKVLRDMNVKESPGRCYSRPVVIFNFANSSYVQYGQLGEDNEILLGNHRTEECQLPSLKIFIAGNSAYEYVDYLFKRMIDLS
Towne         ASCVTINQTSVKVLRDMNVKESPGRCYSRPVVIFNFANSSYVQYGQLGEDNEILLGNHRTEECQLPSLKIFIAGNSAYEYVDYLFKRMIDLS
BE/17/2010    ASCVTINQTSVKVLRDMNVKESPGRCYSRPVVIFNFANSSYVQYGQLGEDNEILLGNHRTEECQLPSLKIFIAGNSAYEYVDYLFKRMIDLS
BE/48/2011    ASCVTINQTSVKVLRDMNVKESPGRCYSRPVVIFNFANSSYVQYGQLGEDNEILLGNHRTEECQLPSLKIFIAGNSAYEYVDYLFKRMIDLS
TB40/E        ASCVTINQTSVKVLRDMNVKESPGRCYSRPVVIFNFANSSYVQYGQLGEDNEILLGNHRTEECQLPSLKIFIAGNSAYEYVDYLFKRMIDLS
BE/5/2011     ASCVTINQTSVKVLRDMNVKESPGRCYSRPVVIFNFANSSYVQYGQLGEDNEILLGNHRTEECQLPSLKIFIAGNSAYEYVDYLFKRMIDLS
BE/32/2011    ASCVTINQTSVKVLRDMNVKESPGRCYSRPVVIFNFANSSYVQYGQLGEDNEILLGNHRTEECQLPSLKIFIAGNSAYEYVDYLFKRMIDLS
C327A         ASCVTINQTSVKVLRDMNVKESPGRCYSRPVVIFNFANSSYVQYGQLGEDNEILLGNHRTEECQLPSLKIFIAGNSAYEYVDYLFKRMIDLS
C178A         ASCVTINQTSVKVLRDMNVKESPGRCYSRPVVIFNFANSSYVQYGQLGEDNEILLGNHRTEECQLPSLKIFIAGNSAYEYVDYLFKRMIDLS
BE/38/2011    ASCVTINQTSVKVLRDMNVKESPGRCYSRPVVIFNFANSSYVQYGQLGEDNEILLGNHRTEECQLPSLKIFIAGNSAYEYVDYLFKRMIDLS
HAN12         ASCVTINQTSVKVLRDMNVKESPGRCYSRPVVIFNFANSSYVQYGQLGEDNEILLGNHRTEECQLPSLKIFIAGNSAYEYVDYLFKRMIDLS
BE/13/2012    ASCVTINQTSVKVLRDMNVKESPGRCYSRPVVIFNFANSSYVQYGQLGEDNEILLGNHRTEECQLPSLKIFIAGNSAYEYVDYLFKRMIDLS
5510-E115     ASCVTINQTSVKVLRDMNVKESPGRCYSRPVVIFNFANSSYVQYGQLGEDNEILLGNHRTEECQLPSLKIFIAGNSAYEYVDYLFKRMIDLS
BE/22/2010    ASCVTINQTSVKVLRDMNVKESPGRCYSRPVVIFNFANSSYVQYGQLGEDNEILLGNHRTEECQLPSLKIFIAGNSAYEYVDYLFKRMIDLS
TR            ASCVTINQTSVKVLRDMNVKDSPGRCYSRPVVIFNFANSSYVQYGQLGEDNEILLGNHRTEECQLPSLKIFIAGNSAYEYVDYLFKRMIDLS
BE/4/2010     ASCVTINQTSVKVLRDMNVKESPGRCYSRPVVIFNFANSSYVQYGQLGEDNEILLGNHRTEECQLPSLKIFIAGNSAYEYVDYLFKRMIDLS
JP            ASCVTINQTSVKVLRDMNVKESPGRCYSRPVVIFNFANSSYVQYGQLGEDNEILLGNHRTEECQLPSLKIFIAGNSAYEYVDYLFKRMIDLS
HAN19         ASCVTINQTSVKVLRDMNVKESPGRCYSRPVVIFNFANSSYVQYGQLGEDNEILLGNHRTEECQLPSLKIFIAGNSAYEYVDYLFKRMIDLS
UKNEQAS1      ASCVTINQTSVKVLRDMTIKDSTGRCYSRPVVIFTFANSSHVQYGQLGEDNEILLGNHRTEECQLPSLKIFIAGNSAYEYVDYLFKRMIDLS
S3            ASCVTINQTSVKVLRDMTIKDSTGRCYSRPVVIFTFANSSHVQYGQLGEDNEILLGNHRTEECQLPSLKIFIAGNSAYEYVDYLFKRMIDLS
consensus>70  ASCVtINQTSVKVLRDMn!K#SpGRCYSRPVVIFnFaNSSyVQYGQLGEDNEILLGNHRTEECQlPSLKIFIAGNSAYEYVDYLFKRMIDLS

             640       650       660       670       680       690       700       710       720
HAN13         SISTVDSMIALDIDPLENTDFRVLELYSQKELRSSNVFDLEEIMREFNSYKQRVKYVEDKVVDPLPPYLKGLDDLMSGLGAA.
BE/6/2011     SISTVDSMIALDIDPLENTDFRVLELYSQKELRSSNVFDLEEIMREFNSYKQRVKYVEDKVVDPLPPYLKGLDDLMSGLGAA.
BE/37/2011    SISTVDSMIALDIDPLENTDFRVLELYSQKELRSSNVFDLEEIMREFNSYKQRVKYVEDKVVDPLPPYLKGLDDLMSGLGAA.
VR1814        SISTVDSMIALDIDPLENTDFRVLELYSQKELRSSNVFDLEEIMREFNSYKQRVKYVEDKVVDPLPPYLKGLDDLMSGLGAA.
HAN1          SISTVDSMIALDIDPLENTDFRVLELYSQKELRSSNVFDLEEIMREFNSYKQRVKYVEDKVVDPLPPYLKGLDDLMSGLGAA.
CO76A         SISTVDSMIALDIDPLENTDFRVLELYSQKELRSSNVFDLEEIMREFNSYKQRVKYVEDKVVDPLPPYLKGLDDLMSGLGAA.
HAN3          SISTVDSMIALDIDPLENTDFRVLELYSQKELRSSNVFDLEEIMREFNSYKQRVKYVEDKVVDPLPPYLKGLDDLMSGLGAA.
BE/42/2011    SISTVDSMIALDIDPLENTDFRVLELYSQKELRSSNVFDLEEIMREFNSYKQRVKYVEDKVVDPLPPYLKGLDDLMSGLGAAG
JHC           SISTVDSMIALDIDPLENTDFRVLELYSQKELRSSNVFDLEEIMREFNSYKQRVKYVEDKVVDPLPPYLKGLDDLMSGLGAA.
BE/13/2010    SISTVDSMIALDIDPLENTDFRVLELYSQKELRSSNVFDLEEIMREFNSYKQRVKYVEDKVVDPLPPYLKGLDDLMSGLGAA.
BE/35/2011    SISTVDSMIALDIDPLENTDFRVLELYSQKELRSSNVFDLEEIMREFNSYKQRVKYVEDKVVDPLPPYLKGLDDLMSGLGAA.
Toledo        SISTVDSMIALDIDPLENTDFRVLELYSQKELRSSNVFDLEEIMREFNSYKQRVKYVEDKVVDPLPPYLKGLDDLMSGLGAA.
C354A         SISTVDSMIALDIDPLENTDFRVLELYSQKELRSSNVFDLEEIMREFNSYKQRVKYVEDKVVDPLPPYLKGLDDLMSGLG...
C359A         SISTVDSMIALDIDPLENTDFRVLELYSQKELRSSNVFDLEEIMREFNSYKQRVKYVEDKVVDPLPPYLKGLDDLMSGLG...
BE/15/2011    SISTVDSMIALDIDPLENTDFRVLELYSQKELRSSNVFDLEEIMREFNSYKQRVKYVEDKVVDPLPPYLKGLDDLMSGLGAAG
HAN38         SISTVDSMIALDIDPLENTDFRVLELYSQKELRSSNVFDLEEIMREFNSYKQRVKYVEDKVVDPLPPYLKGLDDLMSGLGAAG
AD169         SISTVDSMIALDIDPLENTDFRVLELYSQKELRSSNVFDLEEIMREFNSYKQRVKYVEDKVVDPLPPYLKGLDDLMSGLGA..
6397          SISTVDSMIALDIDPLENTDFRVLELYSQKELRSSNVFDLEEIMREFNSYKQRVKYVEDKVVDPLPPYLKGLDDLMSGLGA..
BE/11/2010    SISTVDSMIALDIDPLENTDFRVLELYSQKELRSSNVFDLEEIMREFNSYKQRVKYVEDKVVDPLPPYLKGLDDLMSGLGA..
C336A         SISTVDSMIALDIDPLENTDFRVLELYSQKELRSSNVFDLEEIMREFNSYKQRVKYVEDKVVDPLPPYLKGLDDLMSGLGA..
HAN16         SISTVDSMIALDIDPLENTDFRVLELYSQKELRSSNVFDLEEIMREFNSYKQRVKYVEDKVVDPLPPYLKGLDDLMSGLGA..
BE/16/2012    SISTVDSMIALDIDPLENTDFRVLELYSQKELRSSNVFDLEEIMREFNSYKQRVKYVEDKVVDPLPPYLKGLDDLMSGLGA..
BE/15/2012    SISTVDSMIALDIDPLENTDFRVLELYSQKELRSSNVFDLEEIMREFNSYKQRVKYVEDKVVDPLPPYLKGLDDLMSGLGA..
C338A         SISTVDSMIALDIDPLENTDFRVLELYSQKELRSSNVFDLEEIMREFNSYKQRVKYVEDKVVDPLPPYLKGLDDLMSGLG...
PAV21         SISTVDSMIALDIDPLENTDFRVLELYSQKELRSSNVFDLEEIMREFNSYKQRVKYVEDKVVDPLPPYLKGLDDFMSGLGAA.
C325A         SISTVDSMIALDIDPLENTDFRVLELYSQKELRSSNVFDLEEIMREFNSYKQRVKYVEDKVVDPLPPYLKGLDDLMSGLGA..
BE/4/2012     SISTVDSMIALDIDPLENTDFRVLELYSQKELRSSNVFDLEEIMREFNSYKQRVKYVEDKVVDPLPPYLKGLDDLMSGLGA..
HAN           SISTVDSMIALDIDPLENTDFRVLELYSQKELRSSNVFDLEEIMREFNSYKQRVKYVEDKVVDPLPPYLKGLDDLMSGLGA..
7             SISTVDSMIALDIDPLENTDFRVLELYSQKELRSSNVFDLEEIMREFNSYKQRVKYVEDKVVDPLPPYLKGLDDLMSGLGA..
BE/19/2011    SISTVDSMIALDIDPLENTDFRVLELYSQKELRSSNVFDLEEIMREFNSYKQRVKYVEDKVVDPLPPYLKGLDDLMSGLG...
C194A         SISTVDSMIALDIDPLENTDFRVLELYSQKELRSSNVFDLEEIMREFNSYKQRVKYVEDKVVDPLPPYLKGLDDLMSGLGAA.
BE/5/2012     SISTVDSMIALDIDPLENTDFRVLELYSQKELRSSNVFDLEEIMREFNSYKQRVKYVEDKVVDPLPPYLKGLDDLMSGLGAA.
CZ/2/2012     SISTVDSMIALDIDPLENTDFRVLELYSQKELRSSNVFDLEEIMREFNSYKQRVKYVEDKVVDPLPPYLKGLDDLMSGLGAA.
C128A         SISTVDSMIALDIDPLENTDFRVLELYSQKELRSSNVFDLEEIMREFNSYKQRVKYVEDKVVDPLPPYLKGLDDLMSGLG...
HAN2          SISTVDSMIALDIDPLENTDFRVLELYSQKELRSSNVFDLEEIMREFNSYKQRVKYVEDKVVDPLPPYLKGLDDLMSGLG...
CO82A         SISTVDSMIALDIDPLENTDFRVLELYSQKELRSSNVFDLEEIMREFNSYKQRVKYVEDKVVDPLPPYLKGLDDLMSGLG...
Merlin        SISTVDSMIALDIDPLENTDFRVLELYSQKELRSSNVFDLEEIMREFNSYKQRVKYVEDKVVDPLPPYLKGLDDLMSGLG...
BE/14/2011    SISTVDSMIALDIDPLENTDFRVLELYSQKELRSSNVFDLEEIMREFNSYKQRVKYVEDKVVDPLPPYLKGLDDLMSGLG...
BE/27/2010    SISTVDSMIALDIDPLENTDFRVLELYSQKELRSSNVFDLEEIMREFNSYKQRVKYVEDKVVDPLPPYLKGLDDLMSGLG...
Towne_varL    SISTVDSMIALDIDPLENTDFRVLELYSQKELRSSNVFDLEEIMREFNSYKQRVKYVEDKVVDPLPPYLKGLDDLMSGLG...
3157          SISTVDSMIALDIDPLENTDFRVLELYSQKELRSSNVFDLEEIMREFNSYKQRVKYVEDKVVDPLPPYLKGLDDLMSGLG...
Towne         SISTVDSMIALDIDPLENTDFRVLELYSQKELRSSNVFDLEEIMREFNSYKQRVKYVEDKVVDPLPPYLKGLDDLMSGLG...
BE/17/2010    SISTVDSMIALDIDPLENTDFRVLELYSQKELRSSNVFDLEEIMREFNSYKQRVKYVEDKVVDPLPPYLKGLDDLMSGLG...
BE/48/2011    SISTVDSMIALDIDPLENTDFRVLELYSQKELRSSNVFDLEEIMREFNSYKQRVKYVEDKVVDPLPPYLKGLDDLMSGLG...
TB40/E        SISTVDSMIALDIDPLENTDFRVLELYSQKELRSSNVFDLEEIMREFNSYKQRVKYVEDKVVDPLPPYLKGLDDLMSGLG...
BE/5/2011     SISTVDSMIALDIDPLENTDFRVLELYSQKELRSSNVFDLEEIMREFNSYKQRVKYVEDKVVDPLPPYLKGLDDLMSGLG...
BE/32/2011    SISTVDSMIALDIDPLENTDFRVLELYSQKELRSSNVFDLEEIMREFNSYKQRVKYVEDKVVDPLPPYLKGLDDLMSGLG...
C327A         SISTVDSMIALDIDPLENTDFRVLELYSQKELRSSNVFDLEEIMREFNSYKQRVKYVEDKVVDPLPPYLKGLDDLMSGLG...
C178A         SISTVDSMIALDIDPLENTDFRVLELYSQKELRSSNVFDLEEIMREFNSYKQRVKYVEDKVVDPLPPYLKGLDDLMSGLG...
BE/38/2011    SISTVDSMIALDIDPLENTDFRVLELYSQKELRSSNVFDLEEIMREFNSYKQRVKYVEDKVVDPLPPYLKGLDDLMSGLG...
HAN12         SISTVDSMIALDIDPLENTDFRVLELYSQKELRSSNVFDLEEIMREFNSYKQRVKYVEDKVVDPLPPYLKGLDDLMSGLG...
BE/13/2012    SISTVDSMIALDIDPLENTDFRVLELYSQKELRSSNVFDLEEIMREFNSYKQRVKYVEDKVVDPLPPYLKGLDDLMSGLG...
5510-E115     SISTVDSMIALDIDPLENTDFRVLELYSQKELRSSNVFDLEEIMREFNSYKQRVKYVEDKVVDPLPPYLKGLDDLMSGLG...
BE/22/2010    SISTVDSMIALDIDPLENTDFRVLELYSQKELRSSNVFDLEEIMREFNSYKQRVKYVEDKVVDPLPPYLKGLDDLMSGLG...
TR            SISTVDSMIALDIDPLENTDFRVLELYSQKELRSSNVFDLEEIMREFNSYKQRVKYVEDKVVDPLPPYLKGLDDLMSGL....
BE/4/2010     SISTVDSMIALDIDPLENTDFRVLELYSQKELRSSNVFDLEEIMREFNSYKQRVKYVEDKVVDPLPPYLKGLDDLMSGLG...
JP            SISTVDSMIALDIDPLENTDFRVLELYSQKELRSSNVFDLEEIMREFNSYKQRVKYVEDKVVDPLPPYLKGLDDLMSGLG...
HAN19         SISTVDSMIALDIDPLENTDFRVLELYSQKELRSSNVFDLEEIMREFNSYKQRVKYVEDKVVDPLPPYLKGLDDLMSGLG...
UKNEQAS1      SISTVDSMIALDIDPLENTDFRVLELYSQKELRSSNVFDLEEIMREFNSYKQRVKYVEDKVVDPLPPYLKGLDDLMSGLG...
S3            SISTVDSMIALDIDPLENTDFRVLELYSQKELRSSNVFDLEEIMREFNSYKQRVKYVEDKVVDPLPPYLKGLDDLMSGLG...
consensus>70  SISTVDSMIALDIDPLENTDFRVLELYSQKELRSSNVFDLEEIMREFNSYKQRVKYVEDKVVDPLPPYLKGLDDlMSGLg...
```
