# Supplementary material for: Crystal Structure of the Human Cytomegalovirus Glycoprotein B
Source: PLoS Pathog. 2015 Oct 20;11(10):e1005227. doi: 10.1371/journal.ppat.1005227 (PMC4617298; doi:10.1371/journal.ppat.1005227)
Supplement: S1 Table — Residues used in aligning individual domains of the three homologues are listed. RMSDs between HSV-1 and HCMV gB domains are listed under HSV-1. RMSDs between EBV and HCMV gB domains are listed under EBV. Pymol (http://www.pymol.org) was used to calculate RMSDs. (PDF) [file ppat.1005227.s008.pdf]

Domain		HSV-1	HCMV	EBV
**DI**	Chain B			
	Residues	154-363	133-343	89-294
	RMSD	2.002		1.587
**DII**	Chain B			
	Residues	142-153, 364-476	121-132, 344-438	7-88, 295-390
	RMSD	0.833		0.991
**DIII**	Chain B			
	Residues	117-133, 500-572, 661-669		52-68, 455-527, 617-624
	RMSD	1.396		1.015
**DIV**	Chain B			
	Residues	111-116, 573-660	87-95, 549-637	42-51, 528-616
	RMSD	1.354		0.985
**DV**	Trimer			
	Residues	670-725	647-698	625-679
	RMSD	3.286		2.334
